# A Probabilistic Result on Impulsive Noise Reduction in Topological Data Analysis through Group Equivariant Non-Expansive Operators

**DOI:** 10.3390/e25081150

**Published:** 2023-07-31

**Authors:** Patrizio Frosini, Ivan Gridelli, Andrea Pascucci

**Affiliations:** Department of Mathematics, University of Bologna, 40126 Bologna, Italy; ivangridelli@gmail.com (I.G.); andrea.pascucci@unibo.it (A.P.)

**Keywords:** GENEO, impulsive noise, persistence diagram, persistent homology, machine learning, 55N31, 62R40, 60-08, 65D18, 68T09, 68U05

## Abstract

In recent years, group equivariant non-expansive operators (GENEOs) have started to find applications in the fields of Topological Data Analysis and Machine Learning. In this paper we show how these operators can be of use also for the removal of impulsive noise and to increase the stability of TDA in the presence of noisy data. In particular, we prove that GENEOs can control the expected value of the perturbation of persistence diagrams caused by uniformly distributed impulsive noise, when data are represented by *L*-Lipschitz functions from R to R.

## 1. Introduction

In the last thirty years, Topological Data Analysis (TDA) developed as a useful mathematical theory for analyzing data [1], benefiting from the dimensionality reduction guaranteed by topology [2,3,4]. Topology can construct low-dimensional representations of high-dimensional manifolds of data, thereby reducing the dimensionality of the parameter space and ultimately reducing the learning cost of machine learning models. One of the main tools in TDA is the concept of the persistence diagram, which is a collection of points in the real plane that describes the homological changes of the sublevel sets of suitable continuous functions. These changes give important information about the data of interest, focusing on some of their most relevant properties. Persistence diagrams can be used in the presence of noise, since a well-known stability theorem states that these topological descriptors change in a controlled way when we know that the functions expressing the filtrations we are interested in change in a controlled way with respect to the sup-norm. More precisely, the sup-norm distance between two functions is an upper bound for the bottleneck distance between the corresponding persistence diagrams [5]. Furthermore, $L^{p}$-stability of persistence diagrams with respect to the sup-norm have been proved in [6]. Unfortunately, in many applications, the sup-norm of the noise is not guaranteed to be small, and hence these results cannot be directly applied. In particular, these results cannot be directly used when data are represented by functions from R to R and affected by impulsive noise, i.e., noise that carries a sudden sharp change of short duration, when the variable is seen as a time. It is indeed well known that persistence diagrams can completely change when the data are subject to impulsive noise.

Analogously, in the discrete setting of TDA, the presence of outliers in cloud points can drastically affect the corresponding persistence diagrams. The problem of managing outliers has been studied by several authors with different techniques. In [7], an approach based on confidence sets has been introduced. A method inspired by the *k*-nearest neighbors regression and the local median filtering has been used in [8], while the concept of the bagplot has been applied in [9]. In [10], an approach based on reproducing kernels has been proposed.

In our paper, we start exploring a different probabilistic approach, in the topological setting. The main idea is that studying the properties of data should primarily be based on the analysis of the observers that are asked to examine the data, since we cannot ignore that different observers can differently judge the same data. This approach has been initially proposed in [11] and requires both the definition of the space of group equivariant non-expansive operators (GENEOs) and the development of geometrical techniques to move around in this space [12,13]. In other words, in this model we should not wonder how we have to manage the data but rather how we have to manage the observers (i.e., GENEOs) analyzing the data. As a first step in this direction, in this paper we show how GENEOs could be used to obtain stability of persistence diagrams of 1D signals in the presence of impulsive noise. For the use of such operators in the comparison of 1D signals under the action of several transformation groups, we refer the interested reader to the paper [14]. This type of signal is important in many applications, such as those concerning EEG data and time series.

GENEOs have been studied in [14] as a new tool in TDA, since they allow for an extension of the theory that is not invariant under the action of every homeomorphism of the considered domain. This is important in applications where the invariance group is not the group of all homeomorphisms, such as the ones concerning shape comparison. Interestingly, GENEOs are also deeply related to the foliation method used to define the matching distance in two-dimensional persistent homology [15,16] and can be seen as a theoretical bridge between TDA and Machine Learning [11]. Furthermore, these operators make available lower bounds for the natural pseudo-distance $d_{G}\left(\varphi_{1},\varphi_{2}\right):=\underset{g\in G}{\inf}{∥\varphi_{1}-\varphi_{2}\circ g∥}_{\infty }$, associated with a group *G* of self-homeomorphisms of the domain of the signals $\varphi_{1},\varphi_{2}$ [14]. For general information about the interest in the theory of GENEOs and its applications, we refer the reader to [17].

In our paper, we prove that GENEOs can control the expected value of the perturbation of persistence diagrams caused by uniformly distributed impulsive noise when data are represented by *L*-Lipschitz functions from R to R. In order to do that, we choose a “mother” bump function ψ, i.e., a continuous function that is non-negative, upper bounded by 1, and compactly supported, and we assume that our noise is made up of finite bumps each obtained by translating, heightening, and/or widening ψ. The function $\hat{\varphi }=\varphi +R$ represents the corrupted data, where the noise is given by the function $R=\sum_{i=1}^{k}a_{i}\psi \left(b_{i}\left(x-c_{i}\right)\right)$ for some $a_{i},b_{i},c_{i}\in R$, with $b_{i}>0$, and some positive integer *k*.

In this situation, trying to use a convolution to approximate our starting data is not effective, because even if it does contract the bumps, it does not cut them and hence does not improve the sup-norm distance from the original data. A classical approach for the removal of impulsive noise is the one of using a median filter [18]. Although this approach would be quite efficient in the discrete case, let us remark that in our setting, we are considering continuous functions. The median for the continuous case is defined as the interval of those values *m* such that

$$\int_{-\infty }^{m}f\left(x\right)dx=\int_{m}^{+\infty }f\left(x\right)dx,$$

where *f* is a density of probability. However, this operator is not stable: a small alteration in the starting function could lead to a significant change in the median.

The operators we consider in this paper are $F^{\delta }\left(\varphi \right)\left(x\right)=\max\left(\varphi \left(x-\delta \right),\varphi \left(x+\delta \right)\right)$ and $F_{\varepsilon }\left(\varphi \right)\left(x\right)=\min\left(\varphi \left(x-\varepsilon \right),\varphi \left(x+\varepsilon \right)\right)$. The main idea is that $F^{\delta }$ removes the noise “directed downwards” and $F_{\varepsilon }$ the noise “directed upwards”, and hence, their composition should be able to eliminate all the bumps. These operators are GENEOs with respect to isometries of the real line. We prove that moving in the space of GENEOs by taking suitable values for ε and δ, we can get quite close to restoring the original function φ, depending on how the bumps are positioned. The closer the bumps are to being Dirac delta functions and the further they are from each other, the better our approximation can be. On the ground of this result, we finally obtain an estimate of the expected value $E(∥F^{\delta }\circ F_{\varepsilon }\left(\hat{\varphi }\right)-\varphi {∥}_{\infty })$.

We conclude this introduction by observing that, while the operators we use in this paper are elementary, the theory of GENEOs makes available procedures to learn more complex operators by combining simple GENEOs through techniques belonging to the rising field of Geometric Deep Learning [11,12,13]. For this reason, we hope that our results can not only illustrate a practical use of some particular GENEOs but also be a first step towards the application of Machine Learning for increasing the stability of TDA in the presence of noise.

The paper is structured as follows. In Section 2, the mathematical background is laid. The case we consider and our notations are explained in Section 3. In Section 4, we prove the results that are needed in order to demonstrate our main results. In Section 5, the main theorems giving us probabilistic upper bounds are formulated. In Section 6, some examples and experiments are presented in order to better illustrate the use of our results. A brief discussion concludes the paper.

## 2. Mathematical Setting

In this section, we will recall some basic concepts we will use in this paper.

To avoid misunderstandings about pairs and intervals, we will use the symbols (u,v), [u,v], and ]u,v[ to denote a pair, a closed interval, and an open interval, respectively.

### 2.1. Representing Data as Real Functions

Let us consider a set Φ of bounded functions from a set *X* to R, which will represent the data we wish to take into account (e.g., functions describing sounds). We shall call Φ the set of *admissible measurements* on *X*. We endow Φ with the topology induced by the sup-norm ${∥\cdot ∥}_{\infty }$ and the corresponding distance $D_{\Phi }$. A pseudo-metric $D_{X}$ can be defined on *X* by setting $D_{X}\left(x_{1},x_{2}\right)=\underset{\varphi \in \Phi }{\sup}\left|\varphi \left(x_{1}\right)-\varphi \left(x_{2}\right)\right|$ for every $x_{1},x_{2}\in X$. We recall that a pseudo-metric on a set *X* is a distance *d* without the property d(x,y)=0⇒x=y (in other words, we do not have the so-called “identity of indiscernibles”). We will consider the topological space $\left(X,\tau_{D_{X}}\right)$ where $\tau_{D_{X}}$ is the topology induced by $D_{X}$. A base for this topology is given by the set of open balls {B(x,r):x∈X,r∈R}, where $B\left(x,r\right):=\{x^{\prime }\in X:D_{X}\left(x,x^{\prime }\right)<r\}$. The choice of this topology makes every function in Φ a continuous functions. As shown in [11], this fact enables us to use persistence diagrams in the study of Φ.

### 2.2. GENEOs as Operators Acting on Data

We are interested in considering transformations of data. Let ${\mathrm{Homeo}}_{\Phi }\left(X\right)$ be the group of Φ-preserving homeomorphisms from *X* to *X* with respect to the topology $\tau_{D_{X}}$, meaning that every *g* in ${\mathrm{Homeo}}_{\Phi }\left(X\right)$ is a homeomorphism of *X* such as both φ∘g and $\varphi \circ g^{-1}$ belong to Φ for every φ in Φ. Let *G* be a subgroup of ${\mathrm{Homeo}}_{\Phi }\left(X\right)$. *G* represents the set of transformations on data for which we will require equivariance to be respected.

Under the previously stated assumptions, we call the ordered pair (Φ,G) a *perception pair*. We can now introduce the concept of GENEO.

**Definition 1.**  *Let (Φ,G) and (Ψ,H) be perception pairs and assume that a homomorphism T:G→H is given. A function F:Φ→Ψ is called a Group Equivariant Non-Expansive Operator (GENEO) from (Φ,G) to (Ψ,H) with respect to T if the following properties hold:*

*1.*  *(Group Equivariance) F(φ∘g)=F(φ)∘T(g) for every φ∈Φ,g∈G;*
*2.*  *(Non-Expansivity) $D_{\Psi }\left(F\left(\varphi_{1}\right),F\left(\varphi_{2}\right)\right)\leq D_{\Phi }\left(\varphi_{1},\varphi_{2}\right)$ for every $\varphi_{1},\varphi_{2}\in \Phi$.*

Let us now consider the set $F_{\mathrm{all}}$ of all GENEOs from (Φ,G) to (Ψ,H) with respect to T:G→H. The space $F_{\mathrm{all}}$ is endowed with the extended pseudo-metric $D_{F_{\mathrm{all}}}$, defined by setting $D_{F_{\mathrm{all}}}\left(F_{1},F_{2}\right)=\sup_{\varphi \in \Phi }D_{\Psi }\left(F_{1}\left(\varphi \right),F_{2}\left(\varphi \right)\right)$ for every $F_{1},F_{2}\in F_{\mathrm{all}}$. The word *extended* refers to the possibility that $D_{F_{\mathrm{all}}}$ takes an infinite value.

The following result can be proven [11]:

**Theorem 1.**  *If $\left(\Phi ,D_{\Phi }\right)$,$\left(\Psi ,D_{\Psi }\right)$ are compact and convex then the metric space $\left(F_{\mathrm{all}},D_{F_{\mathrm{all}}}\right)$ is compact and convex.*

If a non-empty set $F\subseteq F_{\mathrm{all}}$ is fixed, we can define the following pseudo-distance $D_{F,\Phi }$ on Φ:

**Definition 2.**  *For any $\varphi_{1},\varphi_{2}$ in Φ we set*

$$D_{F,\Phi }\left(\varphi_{1},\varphi_{2}\right)=\underset{F\in F}{\sup}{∥F\left(\varphi_{1}\right)-F\left(\varphi_{2}\right)∥}_{\infty }.$$

This pseudo-distance allows us to compare data by taking into account how agents operate on data. Notice how, if *G* becomes larger, the natural pseudo-distance $d_{G}$ becomes harder to compute but this new pseudo-distance $D_{F,\Phi }$ becomes easier to evaluate. In order to find a lower bound for $D_{F,\Phi }$ it is useful to introduce the notion of persistence diagram.

### 2.3. Persistence Diagrams

We will now recall some basic definitions and results in persistent homology. The interested reader can find more details in [4].

Let us consider an ordered pair (X,φ) where *X* is a topological space and φ:X→R is a continuous function. For any t∈R we can set $X_{t}:=\varphi^{-1}\left(]-\infty ,t]\right)$. If u<v, the inclusion $i_{u,v}:X_{u}\to X_{v}$ induces a homomorphism $i_{u,v}^{k}:H_{k}\left(X_{u}\right)\to H_{k}\left(X_{v}\right)$ between the *k*th homology groups of $X_{u}$ and $X_{v}$. We can define the *kth persistent homology group*, with respect to φ and computed at the point (u,v), as $PH_{k}\left(u,v\right):=i_{u,v}^{k}\left(H_{k}\left(X_{u}\right)\right)$. Moreover, we can define the *kth persistent Betti numbers function*$r_{k}\left(u,v\right)$ as the rank of $PH_{k}\left(u,v\right)$.

The *k*th persistent Betti numbers function can be represented by the *k*th persistence diagram. This diagram is defined as the multi-set of all the ordered pairs $(u_{j},v_{j}),$ where $u_{j}$ and $v_{j}$ are the times of birth and death of the *j*th *k*-dimensional hole in *X*, respectively. We call *time of birth* of a hole the first time at which the homology class appears, and *time of death* the first time at which the homology class merges with an older one. When a hole never dies, we set its time of death equal to *∞*. We also add to this set all points of the form (w,w) for w∈R.

**Figure 1:**
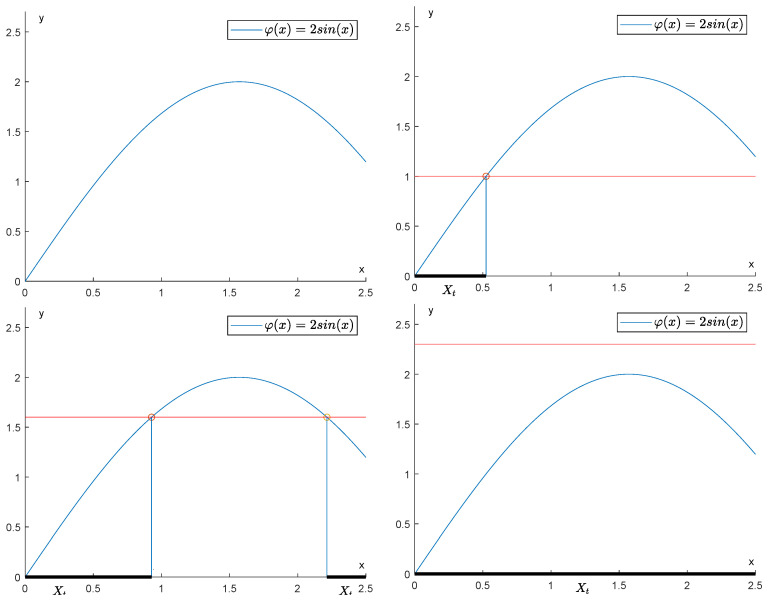
How the sublevel sets change with respect to the filtration induced by φ.

**Figure 2:**
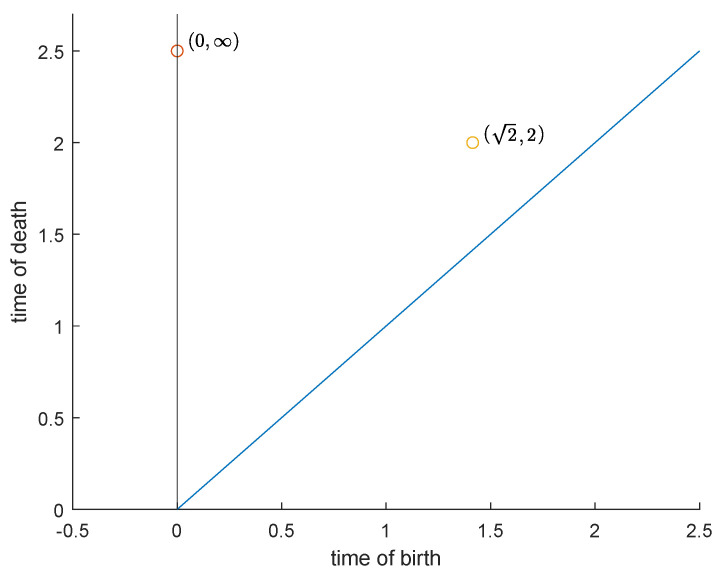
Persistence diagram of the function 2sinx on $\left[0,\frac{3}{4}\pi \right]$. The point (0,∞) describes the existence of a connected component that is born at zero and never dies. The point $\left(\sqrt{2},2\right)$ claims that there is a connected component born at $\sqrt{2}$ that dies (merges with the other one) at 2. The trivial points on the diagonal u=v are not displayed.

In Figure 1 the filtration of the set $X:=\left[0,\frac{3}{4}\pi \right]$ given by the function φ(x)=2sinx is illustrated. In this example, the topology on *X* is the one defined by the space Φ of admissible functions given by all functions asin(x+b) with a,b∈R. The reader can easily check that this topology is the same as the Euclidean topology. The persistence diagram in degree k=0 of the function φ is displayed in Figure 2.

### 2.4. Comparing Persistence Diagrams

Persistence diagrams can be efficiently compared by means of a suitable metric $d_{\mathrm{match}}$. In order to define it, we first define the pseudo-distance

$$\delta \left(\left(x,y\right),\left(x^{\prime },y^{\prime }\right)\right)=\min\left\{\max\left\{\left|x-x^{\prime }\right|,\left|y-y^{\prime }\right|\right\},\max\left\{\left|x-y\right|/2,\left|x^{\prime }-y^{\prime }\right|/2\right\}\right\}$$

for all $\left(x,y\right),\left(x^{\prime },y^{\prime }\right)\in \{\left(x,y\right)\in R$ with x≤y}∪{(x,∞) with x∈R} by agreeing that ∞−y=∞,y−∞=−∞ for y≠∞,∞−∞=0,∞/2=∞,|±∞|=∞,min{∞,c}=c,max{∞,c}=∞.

If two persistence diagrams, $D,D^{\prime }$, are given, we can set

$$d_{\mathrm{match}}\left(D,D^{\prime }\right)=\underset{\sigma \in \Sigma }{\inf}\underset{P\in D}{\sup}\delta \left(P,\sigma \left(P\right)\right)$$

where Σ represents the set of all bijections between the multisets $D,D^{\prime }$. This function is usually called the *bottleneck distance* between *D* and $D^{\prime }$.

For every degree *k* we can now define a new pseudo-metric:

$$D_{F,\Phi }^{\mathrm{match}}\left(\varphi_{1},\varphi_{2}\right)=\underset{F\in F}{\sup}d_{\mathrm{match}}\left(D_{F(\varphi_{1})},D_{F(\varphi_{2})}\right)$$

where $D_{F(\varphi_{1})},D_{F(\varphi_{2})}$ are the persistence diagrams at degree *k* of the functions $F(\varphi_{1})$, $F(\varphi_{2})$, respectively.

In this paper we will limit ourselves to considering data represented as functions from R to R, and we recall that for this kind of data, persistence diagrams are non-trivial only in degree k=0 (i.e., when persistent homology is used to count connected components). For this reason, in the following we will always assume k=0.

## 3. Our Model

In this paper, we will be mainly interested in the set ${\mathrm{Lip}}_{L}$ of all *L*-Lipschitz functions from R to R, for some fixed constant L∈R, and in the set $C^{0}\left(R\right)$ of all functions from R to R that are continuous with respect to the Euclidean topology. We will set X=R and consider the perception pairs $\left({\mathrm{Lip}}_{L},G\right)$, $\left(C^{0}\left(R\right),G\right)$, where *G* is the group of the Euclidean isometries of R.

We will assume our noise originates from a finite number of copies of a “mother” non-negative continuous bump function ψ:R→R, such that $\mathrm{supp}\left(\psi \right)\subseteq ]-\sigma ,\sigma [$ for some σ>0 and ${∥\psi ∥}_{\infty }\leq 1$. We recall that the support of a function is the closure of the set of points where the function is non-zero. After fixing two positive real numbers, η and β, the noise we will be adding is a function *R* belonging to the space $R_{\eta ,\beta }$ that contains the null function and all functions of the form $\sum_{i=1}^{k}a_{i}\psi \left(b_{i}\left(x-c_{i}\right)\right)$, where *k* is a positive integer, and $a_{i},b_{i},c_{i}$ are real numbers such that $|c_{i}-c_{j}|\geq \eta$ for i≠j and $b_{i}\geq \beta$ for every index *i*. For any $R\in R_{\eta ,\beta }$, we define $S\left(R\right):=\overset{k}{\underset{i=1}{⋃}}]c_{i}-\frac{\sigma }{\beta },c_{i}+\frac{\sigma }{\beta }[$ and remark that if x∉S(R), then R(x)=0.

**Figure 3:**
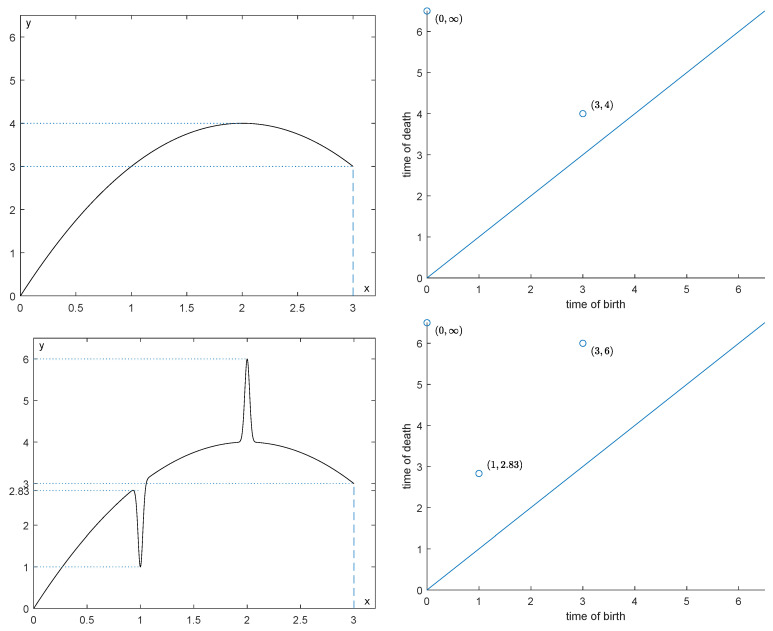
How drastically impulsive noise can influence persistence diagrams.

**Figure 4:**
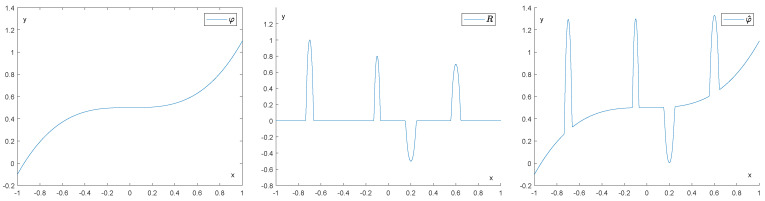
In the figure on the left, we have our original function φ. In the middle we have a noise function R, and in the right figure, we have the corrupted function $\hat{\varphi }:=\varphi +R$.

Figure 3 shows how drastically persistence diagrams can change when the data are subject to impulsive noise. Our purpose will be to recover $\varphi \in {\mathrm{Lip}}_{L}$ as well as possible from the function $\hat{\varphi }=\varphi +R$. An example of such a situation is depicted in Figure 4.

The following result will be of use.

**Proposition 1.**  *Let $F_{1},F_{2}$ be GENEOs from $\left(C^{0}\left(R\right),G\right)$ to $\left(C^{0}\left(R\right),G\right)$ with respect to the trivial homomorphism T=id:G→G. Then $F_{1}\circ F_{2}$ is a GENEO from $\left(C^{0}\left(R\right),G\right)$ to $\left(C^{0}\left(R\right),G\right)$ with respect to T.*

**Proof.**  For every $\varphi \in C^{0}\left(R\right),g\in G$ we have that

$$\begin{array}{cc} F_{1}\circ F_{2}\left(\varphi \circ g\right) & =F_{1}\left(F_{2}\left(\varphi \circ g\right)\right)\\ \; & =F_{1}\left(F_{2}\left(\varphi \right)\circ g\right)\\ \; & =F_{1}\left(F_{2}\left(\varphi \right)\right)\circ g\\ \; & =\left(F_{1}\circ F_{2}\right)\left(\varphi \right)\circ g. \end{array}$$

Therefore, $F_{1}\circ F_{2}$ is *G*-equivariant. Moreover, for any $\varphi_{1},\varphi_{2}\in C^{0}\left(R\right)$

$$\begin{array}{cc} D_{C^{0}\left(R\right)}\left(F_{1}\circ F_{2}\left(\varphi_{1}\right)\right),F_{1}\circ F_{2}\left(\varphi_{2}\right)) & =D_{C^{0}\left(R\right)}\left(F_{1}\left(F_{2}\left(\varphi_{1}\right)\right),F_{1}\left(F_{2}\left(\varphi_{2}\right)\right)\right)\\ \; & \leq D_{C^{0}\left(R\right)}\left(F_{2}\left(\varphi_{1}\right)\right),F_{2}\left(\varphi_{2}\right))\\ \; & \leq D_{C^{0}\left(R\right)}\left(\varphi_{1},\varphi_{2}\right). \end{array}$$

It follows that $F_{1}\circ F_{2}$ is non-expansive. □

## 4. Cutting Off the Noise by GENEOs

We start by introducing two families of GENEOs from $\left(C^{0}\left(R\right),G\right)$ to $\left(C^{0}\left(R\right),G\right)$ with respect to the identical homomorphism.

**Definition 3.**  *Let $\varphi \in {\mathrm{Lip}}_{L}$ and ε>0. For all x∈R we define:*

*1.*  *$F^{\varepsilon }\left(\varphi \right)\left(x\right)=\max\left(\varphi \left(x-\varepsilon \right),\varphi \left(x+\varepsilon \right)\right)$;*
*2.*  *$F_{\varepsilon }\left(\varphi \right)\left(x\right)=\min\left(\varphi \left(x-\varepsilon \right),\varphi \left(x+\varepsilon \right)\right)$.*

**Proposition 2.**  *The maps $F^{\varepsilon }$ and $F_{\varepsilon }$ are GENEOs from $\left(C^{0}\left(R\right),G\right)$ to $\left(C^{0}\left(R\right),G\right)$ with respect to the identical homomorphism.*

**Proof.**  We start by proving that $F^{\varepsilon }$ is *G*-equivariant.

Let *g* be the translation x↦x+k with k∈R, then

$$\begin{array}{cc} F^{\varepsilon }\left(\varphi \circ g\right)\; & =\max\{\varphi ((x+k)-\varepsilon ),\varphi ((x+k)+\varepsilon )\}\\ \; & =\max\{\varphi ((x-\varepsilon )+k),\varphi ((x+\varepsilon )+k)\}\\ \; & =F^{\varepsilon }\left(\varphi \right)\circ g. \end{array}$$

Let *g* be the symmetry x↦−x, then

$$\begin{array}{cc} F^{\varepsilon }\left(\varphi \circ g\right)\; & =\max\{\varphi ((-x)-\varepsilon ),\varphi ((-x)+\varepsilon )\}\\ \; & =\max\{\varphi (-(x+\varepsilon )),\varphi (-(x-\varepsilon ))\}\\ \; & =\max\{\varphi (x+\varepsilon ),\varphi (x-\varepsilon )\}\circ g\\ \; & =F^{\varepsilon }\left(\varphi \right)\circ g. \end{array}$$

Since every isometry in *G* can be written as the composition of a symmetry and a translation, our statement follows.

Furthermore, for any x∈R

$$\begin{array}{cc} \; & |F^{\varepsilon }\left(\varphi_{1}\right)\left(x\right)-F^{\varepsilon }\left(\varphi_{2}\right)\left(x\right)|=\\ \; & =|\max\left\{\varphi_{1}\left(x-\varepsilon \right),\varphi_{1}\left(x+\varepsilon \right)\right\}-\max\left\{\varphi_{2}\left(x-\varepsilon \right),\varphi_{2}\left(x+\varepsilon \right)\right\}|\\ \; & \leq \max\{\left|\varphi_{1}\left(x-\varepsilon \right)-\varphi_{2}\left(x-\varepsilon \right)\right|,\left|\varphi_{1}\left(x+\varepsilon \right)-\varphi_{2}\left(x+\varepsilon \right)\right|\}\\ \; & \leq \max\{∥\varphi_{1}-\varphi_{2}{∥}_{\infty },∥\varphi_{1}-\varphi_{2}{∥}_{\infty }\}\\ \; & =∥\varphi_{1}-\varphi_{2}{∥}_{\infty }. \end{array}$$

It follows that $∥F^{\varepsilon }\left(\varphi_{1}\right)-F^{\varepsilon }\left(\varphi_{2}\right){∥}_{\infty }\leq {∥\varphi_{1}-\varphi_{2}∥}_{\infty }$, and hence $F^{\varepsilon }$ is non-expansive.

We observe that min{a,b}=−max{−a,−b}, and hence $F_{\varepsilon }\left(\varphi \right)=-F^{\varepsilon }\left(-\varphi \right)$ for every $\varphi \in C^{0}\left(R\right)$. Moreover, the map that takes φ to −φ is a GENEO from $\left(C^{0}\left(R\right),G\right)$ to $\left(C^{0}\left(R\right),G\right)$ with respect to the identical homomorphism. Therefore, Proposition 1 ensures that *G*-equivariance and non-expansivity also hold for $F_{\varepsilon }$. □

Since Proposition 1 shows that the composition of GENEOs is still a GENEO, the operator $F^{\delta }\circ F_{\varepsilon }$ is a GENEO from $\left(C^{0}\left(R\right),G\right)$ to $\left(C^{0}\left(R\right),G\right)$ with respect to the identical homomorphism.

We now want to prove that if a function $\hat{\varphi }$ is obtained by adding impulsive noise to a function φ, then the value of

$$∥F^{\delta }\circ F_{\varepsilon }\left(\hat{\varphi }\right){-\varphi ∥}_{\infty }$$

is bounded, and possibly small, provided that δ and ε are chosen appropriately. The main idea is that the operator $F_{\varepsilon }$ cuts the noise “directed upwards” and $F^{\delta }$ cuts the noise “directed downwards”.

In order to proceed, we need two lemmas.

**Lemma 1.**  *Let $R\in C^{0}\left(R\right)$, $\varphi \in {\mathrm{Lip}}_{L}$ for some L∈R, and set $\hat{\varphi }:=\varphi +R$. Then for any ε>0 and δ>0*

(i)

*$-L\varepsilon +F_{\varepsilon }\left(R\right)\leq F_{\varepsilon }\left(\hat{\varphi }\right)-\varphi \leq L\varepsilon +F_{\varepsilon }\left(R\right)$;*

(ii)

*$-L\delta +F^{\delta }\left(R\right)\leq F^{\delta }\left(\hat{\varphi }\right)-\varphi \leq L\delta +F^{\delta }\left(R\right)$;*

(iii)

*$-L\left(\delta +\varepsilon \right)+F^{\delta }\circ F_{\varepsilon }\left(R\right)\leq F^{\delta }\circ F_{\varepsilon }\left(\hat{\varphi }\right)-\varphi \leq L\left(\delta +\varepsilon \right)+F^{\delta }\circ F_{\varepsilon }\left(R\right)$.*

**Proof.**  Since φ is Lipschitz of constant *L*, we have that for any value x∈R |φ(x−ε)−φ(x)|≤Lε and |φ(x+ε)−φ(x)|≤Lε. Therefore,

$$\begin{array}{cc} F_{\varepsilon }\left(\hat{\varphi }\right)\left(x\right) & =F_{\varepsilon }\left(\varphi +R\right)\left(x\right)\\ \; & =\min\{\varphi (x-\varepsilon )+R(x-\varepsilon ),\varphi (x+\varepsilon )+R(x+\varepsilon )\}\\ \; & \leq \min\{\varphi (x)+L\varepsilon +R(x-\varepsilon ),\varphi (x)+L\varepsilon +R(x+\varepsilon )\}\\ \; & =\varphi (x)+L\varepsilon +\min\{R(x-\varepsilon ),R(x+\varepsilon )\}\\ \; & =\varphi \left(x\right)+L\varepsilon +F_{\varepsilon }\left(R\right)\left(x\right). \end{array}$$

Analogously, $F_{\varepsilon }\left(\hat{\varphi }\right)\left(x\right)\geq \varphi \left(x\right)-L\varepsilon +F_{\varepsilon }\left(R\right)\left(x\right)$. The same steps applied to $F^{\delta }$ yield the second statement of the lemma. As for the last claim, we can see that:

$$\begin{array}{cc} \; & F^{\delta }\circ F_{\varepsilon }\left(\hat{\varphi }\right)\left(x\right)\\ \; & =\max\{F_{\varepsilon }\left(\hat{\varphi }\right)\left(x-\delta \right),F_{\varepsilon }\left(\hat{\varphi }\right)\left(x+\delta \right)\}\\ \; & \leq \max\{\varphi \left(x-\delta \right)+L\varepsilon +F_{\varepsilon }\left(R\right)\left(x-\delta \right),\varphi \left(x+\delta \right)+L\varepsilon +F_{\varepsilon }\left(R\right)\left(x+\delta \right)\}\\ \; & \leq \max\{\varphi \left(x\right)+L\delta +L\varepsilon +F_{\varepsilon }\left(R\right)\left(x-\delta \right),\varphi \left(x\right)+L\delta +L\varepsilon +F_{\varepsilon }\left(R\right)\left(x+\delta \right)\}\\ \; & =\varphi \left(x\right)+L\delta +L\varepsilon +\max\{F_{\varepsilon }\left(R\right)\left(x-\delta \right),F_{\varepsilon }\left(R\right)\left(x+\delta \right)\}\\ \; & =\varphi \left(x\right)+L\delta +L\varepsilon +F^{\delta }\circ F_{\varepsilon }\left(R\right)\left(x\right). \end{array}$$

Analogously, we can prove the lower bound. □

Henceforth, we will assume that any summation on an empty set of indexes is the null function.

**Lemma 2.**  *Let $R\in R_{\eta ,\beta }$ and $\lambda \geq \frac{\sigma }{\beta }$. If $\lambda \leq \rho \leq \frac{\eta }{2}-\lambda$ then*

(a)

$F_{\rho }\left(R\right)\left(x\right)=\sum_{a_{i}<0}\left[a_{i}\psi \left(b_{i}\left(x-\rho -c_{i}\right)\right)+a_{i}\psi \left(b_{i}\left(x+\rho -c_{i}\right)\right)\right]\leq 0$

(b)

$F^{\rho }\left(R\right)\left(x\right)=\sum_{a_{i}>0}\left[a_{i}\psi \left(b_{i}\left(x-\rho -c_{i}\right)\right)+a_{i}\psi \left(b_{i}\left(x+\rho -c_{i}\right)\right)\right]\geq 0$

*for all x∈R. Moreover $F_{\rho }\left(R\right),F^{\rho }\left(R\right)\left(x\right)\in R_{2\lambda ,\beta }$.*

**Proof.**  We will suppose without loss of generality that $c_{i}<c_{i+1}$ for all i=1,…,k−1 and $a_{i}\neq 0$ for all indexes *i*.

We want to show that at least one of x−ρ and x+ρ must always be outside of $\overset{k}{\underset{i=1}{⋃}}]c_{i}-\lambda ,c_{i}+\lambda [$. If by contradiction both x−ρ and x+ρ were in the same $]c_{i}-\lambda ,c_{i}+\lambda [$, then we would have ρ<λ, against our hypotheses. Moreover, x−ρ and x+ρ cannot belong to different intervals $]c_{i}-\lambda ,c_{i}+\lambda [$ and $]c_{j}-\lambda ,c_{j}+\lambda [$ for i<j: if by contradiction $x-\rho \in ]c_{i}-\lambda ,c_{i}+\lambda [$ and $x+\rho \in ]c_{j}-\lambda ,c_{j}+\lambda [$ for some i<j, then $2\rho =x+\rho -\left(x-\rho \right)>\left(c_{j}-\lambda \right)-\left(c_{i}+\lambda \right)\geq c_{j}-c_{i}-2\lambda \geq \eta -2\lambda$, and hence we would have $\rho >\frac{\eta }{2}-\lambda$, against our hypotheses.

Since $]c_{i}-\frac{\sigma }{\beta },c_{i}+\frac{\sigma }{\beta }[\subseteq ]c_{i}-\lambda ,c_{i}+\lambda [$, it follows that at least one among the values R(x−ρ) and R(x+ρ) must always be zero. Let us now set $I_{i}^{-}:=]\left(c_{i}-\rho \right)-\lambda ,\left(c_{i}-\rho \right)+\lambda [$ and $I_{i}^{+}:=]\left(c_{i}+\rho \right)-\lambda ,\left(c_{i}+\rho \right)+\lambda [$. These two intervals must be disjoint, since $\left(c_{i}+\rho -\lambda \right)-\left(c_{i}-\rho +\lambda \right)=2\rho -2\lambda \geq 0$.

Let us now consider $\left\{I_{1}^{-},I_{1}^{+},\ldots ,I_{k}^{-},I_{k}^{+}\right\}$. We will now prove that any two distinct elements from this set must be a disjoint. Since we have just proven that $I_{i}^{-}\cap I_{i}^{+}=\emptyset$ for i=1,…,k, the following holds in the case i<j:

1. $I_{i}^{+}\cap I_{j}^{+}=\emptyset$, since $c_{j}+\rho -\lambda -\left(c_{i}+\rho +\lambda \right)\geq c_{j}-c_{i}-2\lambda \geq \eta -2\lambda \geq 0$;
2. $I_{i}^{-}\cap I_{j}^{-}=\emptyset$, since $c_{j}-\rho -\lambda -\left(c_{i}-\rho +\lambda \right)\geq c_{j}-c_{i}-2\lambda \geq \eta -2\lambda \geq 0$;
3. $I_{i}^{+}\cap I_{j}^{-}=\emptyset$, since $c_{j}-\rho -\lambda -\left(c_{i}+\rho +\lambda \right)\geq c_{j}-c_{i}-2\rho -2\lambda \geq \eta -2\left(\frac{\eta }{2}-\lambda \right)-2\lambda =0$;
4. $I_{j}^{+}\cap I_{i}^{-}$, since $c_{j}+\rho -\lambda -\left(c_{i}-\rho +\lambda \right)\geq c_{j}-c_{i}+2\rho -2\lambda \geq \eta +2\lambda -2\lambda \geq 0$.

Let us now fix k∈{1,…,k}.

Since $\mathrm{supp}\left(a_{k}\psi \left(b_{k}\left(x-c_{k}\right)\right)\right)\subseteq ]c_{k}-\lambda ,c_{k}+\lambda [$, then $\mathrm{supp}\left(a_{k}\psi \left(b_{k}\left(x-\rho -c_{k}\right)\right)\right)\subseteq I_{k}^{-}$ and $\mathrm{supp}\left(a_{k}\psi \left(b_{k}\left(x+\rho -c_{k}\right)\right)\right)\subseteq I_{k}^{+}$.

Hence, for any x∈R we have that

$$\begin{array}{cc} F_{\rho }\left(R\right)\left(x\right) & =\min\{R(x-\rho ),R(x+\rho )\}\\ \; & =\left\{\begin{array}{cc} 0 & if\;x\notin \overset{k}{\underset{i=1}{⋃}}\left(I_{i}^{-}\cup I_{i}^{+}\right)\\ a_{j}\psi \left(b_{j}\left(x+\rho -c_{j}\right)\right) & if\;x\in I_{j}^{-}\;\mathrm{and}\;a_{j}<0\\ a_{j}\psi \left(b_{j}\left(x-\rho -c_{j}\right)\right) & if\;x\in I_{j}^{+}\;\mathrm{and}\;a_{j}<0. \end{array}\right. \end{array}$$

This means that

$$F_{\rho }\left(R\right)\left(x\right)=\sum_{a_{i}<0}\left[a_{i}\psi \left(b_{i}\left(x-\rho -c_{i}\right)\right)+a_{i}\psi \left(b_{i}\left(x+\rho -c_{i}\right)\right)\right]\leq 0.$$

Now, given $c_{i}+\rho$ and $c_{j}-\rho$ centers of bumps of $F_{\rho }\left(R\right)$, we have that

$$\left|\left(c_{i}+\rho \right)-\left(c_{j}-\rho \right)\right|\geq \min\left\{\eta -2\rho ,2\rho \right\}\geq 2\lambda .$$

It follows that $F_{\rho }\left(R\right)\in R_{2\lambda ,\beta }$.

By noting that $F^{\rho }\left(R\right)=-F_{\rho }\left(-R\right)$, we obtain the second part of the thesis. □

Let us remark that, in particular, if $\lambda =2\frac{\sigma }{\beta }$, then $F_{\rho }\left(R\right),F^{\rho }\left(R\right)\in R_{4\frac{\sigma }{\beta },\beta }$. We observe that for there to exist a ρ that satisfies the hypotheses of Lemma 2, the inequality η≥4λ must hold.

We are now actually ready to prove a result that will be useful in the remainder of our paper.

**Theorem 2.**  *Given θ,β,L∈R, let $\bar{R}\in R_{\theta ,\beta }$, $\varphi \in {\mathrm{Lip}}_{L}$ and set $\hat{\varphi }:=\varphi +\bar{R}$. If $2\frac{\sigma }{\beta }\leq \varepsilon \leq \frac{\theta }{2}-2\frac{\sigma }{\beta }$ then for any $\frac{\sigma }{\beta }\leq \delta \leq \frac{1}{2}\min\left\{\theta -2\varepsilon ,2\varepsilon \right\}-\frac{\sigma }{\beta }$ the following inequality holds:*

$$∥F^{\delta }\circ F_{\varepsilon }\left(\hat{\varphi }\right){-\varphi ∥}_{\infty }\leq L\left(\varepsilon +\delta \right).$$

**Proof.**  Let x∈R. Firstly, let us notice that the condition $2\frac{\sigma }{\beta }\leq \varepsilon \leq \frac{\theta }{2}-2\frac{\sigma }{\beta }$ implies that $\frac{\sigma }{\beta }\leq \frac{1}{2}\min\left\{\theta -2\varepsilon ,2\varepsilon \right\}-\frac{\sigma }{\beta }$. From Lemma 1, we know that

$$-L\left(\delta +\varepsilon \right)+F^{\delta }\circ F_{\varepsilon }\left(\bar{R}\right)\left(x\right)\leq F^{\delta }\circ F_{\varepsilon }\left(\hat{\varphi }\right)\left(x\right)-\varphi \left(x\right)\leq L\left(\delta +\varepsilon \right)+F^{\delta }\circ F_{\varepsilon }\left(\bar{R}\right)\left(x\right).$$

Let us now prove that $F^{\delta }\circ F_{\varepsilon }\left(\bar{R}\right)\left(x\right)=0$.

By applying Lemma 2 with η:=θ, $\lambda :=2\frac{\sigma }{\beta }$, ρ:=ε and $R:=\bar{R}$, we obtain

$$F_{\varepsilon }\left(\bar{R}\right)\left(x\right)=\sum_{a_{i}<0}\left[a_{i}\psi \left(b_{i}\left(x-\varepsilon -c_{i}\right)\right)+a_{i}\psi \left(b_{i}\left(x+\varepsilon -c_{i}\right)\right)\right]\leq 0.$$

Let us remark that $F_{\varepsilon }\left(\bar{R}\right)\in R_{4\frac{\sigma }{\beta },\beta }$. Moreover,

1. $F^{\delta }\circ F_{\varepsilon }\left(\bar{R}\right)\left(x\right)=\max\left\{F_{\varepsilon }\left(\bar{R}\right)\left(x-\delta \right),F_{\varepsilon }\left(\bar{R}\right)\left(x+\delta \right)\right\}\leq 0$ since both terms are negative;
2. $F^{\delta }\circ F_{\varepsilon }\left(\bar{R}\right)\left(x\right)=F^{\delta }\left(F_{\varepsilon }\left(\bar{R}\right)\right)\left(x\right)\geq 0$.

These inequalities follow from Lemma 2, by setting $\eta :=4\frac{\sigma }{\beta }$, $\lambda :=\frac{\sigma }{\beta }$, ρ:=δ and $R:=F_{\varepsilon }\left(\bar{R}\right)$.

Therefore, we have proved that $\left|F^{\delta }\circ F_{\varepsilon }\left(\hat{\varphi }\right)\left(x\right)-\varphi \left(x\right)\right|\leq L(\delta +\varepsilon$) for any x∈R. It follows that $∥F^{\delta }\circ F_{\varepsilon }\left(\hat{\varphi }\right){-\varphi ∥}_{\infty }\leq L\left(\varepsilon +\delta \right)$. □

Let us remark that Theorem 2 works under the (only) implicit assumption that $\theta \geq 8\frac{\sigma }{\beta }$. In our setting, this should not be restrictive since it means that the noise added is made up of scattered, thin bumps, without any reference to the height of the bumps. This is what we expect when considering additive impulsive noise.

**Corollary 1.**  *Given θ,β,L∈R, let $R\in R_{\theta ,\beta }$, $\varphi \in {\mathrm{Lip}}_{L}$, and set $\hat{\varphi }:=\varphi +R$. If $\theta \geq 8\frac{\sigma }{\beta }$, then*

$${∥F^{\frac{\sigma }{\beta }}\circ F_{2\frac{\sigma }{\beta }}\left(\hat{\varphi }\right)-\varphi ∥}_{\infty }\leq 3L\frac{\sigma }{\beta }.$$

**Proof.**  The claim follows from Theorem 2 by taking $\varepsilon :=2\frac{\sigma }{\beta }$ and $\delta :=\frac{\sigma }{\beta }.$ □

Corollary 1 and the well-known stability of persistence diagrams with respect to the max-norm [5] immediately imply the following result, which is of interest in TDA (the symbol $d_{\mathrm{match}}$ denotes the usual bottleneck distance between persistence diagrams).

**Corollary 2.**  *Given θ,β,L∈R, let $R\in R_{\theta ,\beta }$, $\varphi \in {\mathrm{Lip}}_{L}$, and set $\hat{\varphi }:=\varphi +R$. If $\theta \geq 8\frac{\sigma }{\beta }$, and D and $D^{\prime }$ are the persistence diagrams in degree 0 of the filtering functions φ and $F^{\frac{\sigma }{\beta }}\circ F_{2\frac{\sigma }{\beta }}\left(\hat{\varphi }\right)$, respectively, then*

$$d_{\mathrm{match}}\left(D,D^{\prime }\right)\leq 3L\frac{\sigma }{\beta }.$$

## 5. Our Main Results

We are now ready to prove our main results. We start by recalling a simple statement concerning the probability *p* that any two distinct points in a randomly chosen set of cardinality *k* in an interval of length *ℓ* have a distance greater than η (cf., e.g., [19]). For the reader’s convenience, we report the proof here.

**Lemma 3.**  *Let $X_{1},\ldots ,X_{k}$, with k≥2, be independent random variables, uniformly distributed on the interval [0,ℓ], for some ℓ>0. Let*

M:=min1≤i,j≤ki≠j|Xi−Xj|

*be the minimal distance between two distinct random variables. Then, we have*

$$P\left(M>\eta \right)=\left\{\begin{array}{cc} 1 & \mathrm{if}\;\eta \leq 0,\\ {\left(1-\frac{(k-1)\eta }{\ell }\right)}^{k} & \mathrm{if}\;0<\eta <\frac{\ell }{k-1},\\ 0 & \mathrm{if}\;\eta \geq \frac{\ell }{k-1}. \end{array}\right.$$

**Proof.**  It suffices to consider the case $0<\eta <\frac{\ell }{k-1}$. By symmetry, we have

$$P(M>\eta )=k!\;P(\left(M>\eta \right)\cap \left(X_{1}<X_{2}<\cdots <X_{k}\right))$$

(since $X_{1},\ldots ,X_{k}$ are uniformly distributed)

(1) $$=k!\frac{\mathrm{Leb}(S)}{\ell^{k}}$$

where $S=\{x\in {\left[0,\ell \right]}^{k}|x_{1}<x_{2}-\eta <x_{3}-2\eta <\cdots <x_{k}-\left(k-1\right)\eta \}$, and Leb denotes the Lebesgue measure. Setting $y_{i}=x_{i}-\left(i-1\right)\eta$ for i=1,…,k, we have that $\mathrm{Leb}\left(S\right)=\mathrm{Leb}\left(S^{\prime }\right)$ where

$$S^{\prime }=\left\{y\in {\left[0,\ell -\left(k-1\right)\eta \right]}^{k}|y_{1}<y_{2}<\cdots <y_{k}\right\}.$$

On the other hand, again by symmetry, we have

$$\mathrm{Leb}\left(S^{\prime }\right)=\frac{\mathrm{Leb}({\left[0,\ell -\left(k-1\right)\eta \right]}^{k})}{k!}=\frac{{\left(\ell -\left(k-1\right)\eta \right)}^{k}}{k!}$$

and plugging this last identity into (1) we obtain the thesis. □

We can now prove the following result, concerning the expected value of the error ${∥F^{\frac{\sigma }{\beta }}\circ F_{2\frac{\sigma }{\beta }}\left(\hat{\varphi }\right)-\varphi ∥}_{\infty }$. We remark explicitly that the error is random because it is a function of $c_{1},\ldots ,c_{k}$, which are independent random variables uniformly distributed on the interval [0,ℓ].

**Theorem 3.**  *Let us choose a function $\varphi \in {\mathrm{Lip}}_{L}$, a non-negative continuous function ψ:R→R with ${∥\psi ∥}_{\infty }\leq 1$ and $\mathrm{supp}\left(\psi \right)\subseteq ]-\sigma ,\sigma [$ for some σ>0, two positive numbers β and ℓ, and an integer k≥2. For i=1,…,k, let us fix $a_{i}\in R$ and $b_{i}\geq \beta$, and set $\bar{\alpha }:=\max\left|a_{i}\right|$. Moreover, let $c_{1},\ldots ,c_{k}$ be independent random variables, uniformly distributed on the interval [0,ℓ]. Let us consider the random variable $\hat{\varphi }:=\varphi +R$, where $R\left(x\right):=\sum_{i=1}^{k}a_{i}\psi \left(b_{i}\left(x-c_{i}\right)\right)$ for any x∈R. If $\frac{\sigma }{\beta }<\frac{\ell }{8(k-1)}$, then*

$$E\left({∥F^{\frac{\sigma }{\beta }}\circ F_{2\frac{\sigma }{\beta }}\left(\hat{\varphi }\right)-\varphi ∥}_{\infty }\right)\leq 3L\frac{\sigma }{\beta }+k\bar{\alpha }\left(1-{\left(1-8\frac{\left(k-1\right)}{\ell }\frac{\sigma }{\beta }\right)}^{k}\right).$$

**Proof.**  By setting $\delta =\frac{\sigma }{\beta }$ and $\varepsilon =2\frac{\sigma }{\beta }$ in statement (iii) of Lemma 1, we have that

$${∥F^{\frac{\sigma }{\beta }}\circ F_{2\frac{\sigma }{\beta }}\left(\hat{\varphi }\right)-\varphi ∥}_{\infty }\leq 3L\frac{\sigma }{\beta }+{∥F^{\frac{\sigma }{\beta }}\circ F_{2\frac{\sigma }{\beta }}\left(R\right)∥}_{\infty }.$$

Since the operator $F^{\frac{\sigma }{\beta }}\circ F_{2\frac{\sigma }{\beta }}$ is non-expansive and $F^{\frac{\sigma }{\beta }}\circ F_{2\frac{\sigma }{\beta }}\left(0\right)=0$, it follows that

$${∥F^{\frac{\sigma }{\beta }}\circ F_{2\frac{\sigma }{\beta }}\left(R\right)∥}_{\infty }\leq {∥R∥}_{\infty }\leq k\bar{\alpha }{∥\psi ∥}_{\infty }\leq k\bar{\alpha }.$$

Therefore, ${∥F^{\frac{\sigma }{\beta }}\circ F_{2\frac{\sigma }{\beta }}\left(\hat{\varphi }\right)-\varphi ∥}_{\infty }\leq 3L\frac{\sigma }{\beta }+k\bar{\alpha }$. If we apply Lemma 3 with $\eta =8\frac{\sigma }{\beta }$, we obtain that $R\in R_{8\frac{\sigma }{\beta },\beta }$ with probability $p:={\left(1-8\frac{\left(k-1\right)}{\ell }\frac{\sigma }{\beta }\right)}^{k}$. If $R\in R_{8\frac{\sigma }{\beta },\beta }$, we can apply Theorem 2 by setting $\delta =\frac{\sigma }{\beta }$, $\varepsilon =2\frac{\sigma }{\beta }$ and $\theta =8\frac{\sigma }{\beta }$, and hence we obtain that ${∥F^{\frac{\sigma }{\beta }}\circ F_{2\frac{\sigma }{\beta }}\left(\hat{\varphi }\right)-\varphi ∥}_{\infty }\leq 3L\frac{\sigma }{\beta }$ with probability at least *p*. Since ${∥F^{\frac{\sigma }{\beta }}\circ F_{2\frac{\sigma }{\beta }}\left(\hat{\varphi }\right)-\varphi ∥}_{\infty }\leq 3L\frac{\sigma }{\beta }+k\bar{\alpha }$ in any case, it follows that

$$\begin{array}{cc} E\left({∥F^{\frac{\sigma }{\beta }}\circ F_{2\frac{\sigma }{\beta }}\left(\hat{\varphi }\right)-\varphi ∥}_{\infty }\right) & \leq 3L\frac{\sigma }{\beta }p+\left(3L\frac{\sigma }{\beta }+k\bar{\alpha }\right)\left(1-p\right)\\ \; & =3L\frac{\sigma }{\beta }+k\bar{\alpha }\left(1-p\right). \end{array}$$

□

Theorem 3 and the well-known stability of persistence diagrams with respect to the max-norm [5] immediately imply the following result, which is of interest in TDA (the symbol $d_{\mathrm{match}}$ denotes the usual bottleneck distance between persistence diagrams).

**Corollary 3.**  *Let us make the same assumptions of Theorem 3. Let D and $D^{\prime }$ be the persistence diagrams in degree 0 of the filtering functions φ and $F^{\frac{\sigma }{\beta }}\circ F_{2\frac{\sigma }{\beta }}\left(\hat{\varphi }\right)$, respectively. If $\frac{\sigma }{\beta }<\frac{\ell }{8(k-1)}$, then*

$$E\left(d_{\mathrm{match}}\left(D,D^{\prime }\right)\right)\leq 3L\frac{\sigma }{\beta }+k\bar{\alpha }\left(1-{\left(1-8\frac{\left(k-1\right)}{\ell }\frac{\sigma }{\beta }\right)}^{k}\right).$$

This result shows that the use of suitable GENEOs can make TDA (relatively) stable also in the presence of impulsive noise, under the assumptions we have considered in this paper.

Theorem 3 and Corollary 3 can be easily extended to the case that the number *k* of bumps is a random variable:

**Theorem 4.**  *Let k be a random variable that takes values in the subset S={2,3,…,K} of the positive integers, where we assume that the probability of the integer j∈S is $p_{j}$. Let us choose a function $\varphi \in {\mathrm{Lip}}_{L}$, a non-negative continuous function ψ:R→R with ${∥\psi ∥}_{\infty }\leq 1$ and $\mathrm{supp}\left(\psi \right)\subseteq ]-\sigma ,\sigma [$ for some σ>0, two positive numbers β and ℓ. Let $c_{1}^{k},\ldots ,c_{k}^{k}$ be random variables that, conditioned to k, are independent and uniformly distributed on the interval [0,ℓ]. Moreover, for i=1,…,k, let us fix $a_{i}^{k}\in R$ and $b_{i}^{k}\geq \beta$, and set ${\bar{\alpha }}_{k}:=\max_{i}\left|a_{i}^{k}\right|$. Let us also consider the random variable $\hat{\varphi }:=\varphi +R_{k}$, where $R_{k}\left(x\right):=\sum_{i=1}^{k}a_{i}^{k}\psi \left(b_{i}^{k}\left(x-c_{i}^{k}\right)\right)$ for any x∈R. If $\frac{\sigma }{\beta }<\frac{\ell }{8(K-1)}$, then*

$$E\left({∥F^{\frac{\sigma }{\beta }}\circ F_{2\frac{\sigma }{\beta }}\left(\hat{\varphi }\right)-\varphi ∥}_{\infty }\right)\leq 3L\frac{\sigma }{\beta }+\sum_{j=2}^{K}p_{j}j{\bar{\alpha }}_{j}\left(1-{\left(1-8\frac{\left(j-1\right)}{\ell }\frac{\sigma }{\beta }\right)}^{j}\right).$$

**Proof.**  It follows immediately from Theorem 3. □

**Corollary 4.**  *Let us make the same assumptions of Theorem 4. Let D and $D^{\prime }$ be the persistence diagrams in degree 0 of the filtering functions φ and $F^{\frac{\sigma }{\beta }}\circ F_{2\frac{\sigma }{\beta }}\left(\hat{\varphi }\right)$, respectively. If $\frac{\sigma }{\beta }<\frac{\ell }{8(K-1)}$, then*

$$E\left(d_{\mathrm{match}}\left(D,D^{\prime }\right)\right)\leq 3L\frac{\sigma }{\beta }+\sum_{j=2}^{K}p_{j}j{\bar{\alpha }}_{j}\left(1-{\left(1-8\frac{\left(j-1\right)}{\ell }\frac{\sigma }{\beta }\right)}^{j}\right).$$

**Proof.**  It follows immediately from Corollary 3. □

## 6. Examples and Experiments

We will now validate our approach based on GENEOs by giving two examples and illustrating some experimental results.

### 6.1. Examples

In order to verify how our approach works, we will set $\tau_{n}:=\left(1-\frac{1}{n}\right)2\frac{\sigma }{\beta }+\frac{1}{n}\left(\frac{\theta }{2}-2\frac{\sigma }{\beta }\right)$ and consider the upper bound ${∥F^{\frac{\tau_{n}}{2}}\circ F_{\tau_{n}}\left(\hat{\varphi }\right)-\varphi ∥}_{\infty }\leq \frac{3}{2}L\tau_{n}$, obtained by applying Corollary 1. We observe that $\tau_{n}\geq 2\frac{\sigma }{\beta }$ for every index *n*, and $\lim_{n\to +\infty }\tau_{n}=2\frac{\sigma }{\beta }$. We will examine two examples that use the GENEOs $F^{\frac{\tau_{n}}{2}}\circ F_{\tau_{n}}$ and show how our method based on such operators and the method based on convolutions differ, as for their capability in removing additive impulsive noise. Moreover, we will compare the actual error ${∥F^{\frac{\tau_{n}}{2}}\circ F_{\tau_{n}}\left(\hat{\varphi }\right)-\varphi ∥}_{\infty }$ to its upper bound $\frac{3}{2}L\tau_{n}$, by running several simulations. The convolutions that will be applied in our examples use the functions $T_{h}:R\to R$ defined by setting

$$T_{h}\left(x\right):=\left\{\begin{array}{cc} \frac{h}{2} & if-\frac{1}{h}\leq x\leq \frac{1}{h}\\ 0 & otherwise \end{array}\right.$$

for h>0. We will see that, although the convolution with such functions is also a GENEO, it will not be able to efficiently remove the noise.

Our noise function *R* will be constructed starting from the mother function ψ defined by setting $\psi \left(x\right):=e^{1-\frac{1}{1-x^{2}}}$ for $x\in ]-1,1[$ and ψ(x):=0 for $x\notin ]-1,1[$. Using the notation introduced in the previous sections, we will set σ=1.1, thus satisfying the condition $\mathrm{supp}\left(\psi \right)\subseteq ]-\sigma ,\sigma [$. The impulsive noise will be added in an interval $\left[-\ell ,\ell \;\right]$. The following parameters are considered, with these respective uniform distributions:

- $k∼{\mathrm{Unif}}_{\left\{1,\ldots ,10\right\}}$
- $a_{i}∼{\mathrm{Unif}}_{]-100,100[}$ for i=1,…,k
- $b_{i}∼{\mathrm{Unif}}_{]0,100[}$ for i=1,…,k
- $c_{i}∼{\mathrm{Unif}}_{]-\ell +\frac{\sigma }{\beta },\ell -\frac{\sigma }{\beta }[}$ for i=1,…,k.

We set $\beta :=\min_{i=1,\ldots ,N}b_{i}$, $\bar{\alpha }:=\max_{i=1,\ldots ,N}\left|a_{i}\right|$, and $\eta :=\min_{i\neq j}\left|c_{i}-c_{j}\right|$. After producing random values for the parameters $N,a_{i},b_{i},c_{i}$, our algorithm checks whether $\eta >8\frac{\sigma }{\beta }$; otherwise it generates another set of parameters.

#### 6.1.1. First Example

**Figure 5:**
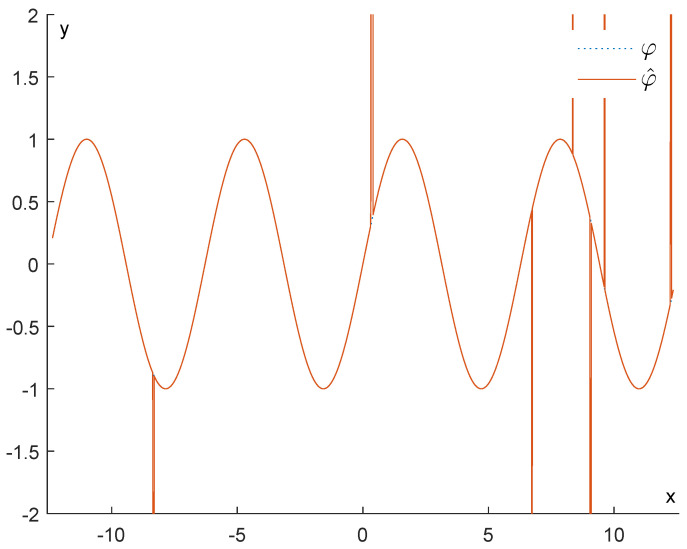
Example 1: Comparison between φ and $\hat{\varphi }$.

Let us consider the function

$$\varphi \left(x\right):=\left\{\begin{array}{cc} \sin x & if-4\pi \leq x\leq 4\pi \\ 0 & otherwise \end{array}\right.$$

for x∈R. We observe that $\varphi \in {\mathrm{Lip}}_{L}$, for L=1. We will add noise in the interval [−4π,4π] (meaning ℓ=4π) and visualize the results in such an interval. Figure 5 illustrates how the function $\hat{\varphi }$ looks compared to φ.

**Figure 6:**
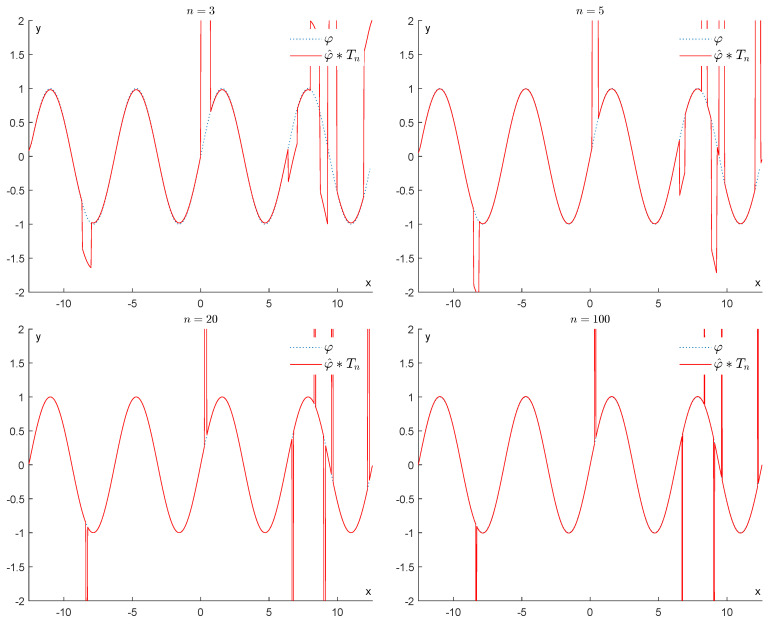
Example 1: Denoising via convolution with $T_{h}$ (h=n).

We will start by considering how well the convolution $\hat{\varphi }*T_{n}$ can approximate the original function φ, when *n* goes from 3 to 100. From Figure 6, it is immediately apparent that the max-norm distance between $\hat{\varphi }*T_{n}$ and φ remains quite large.

**Figure 7:**
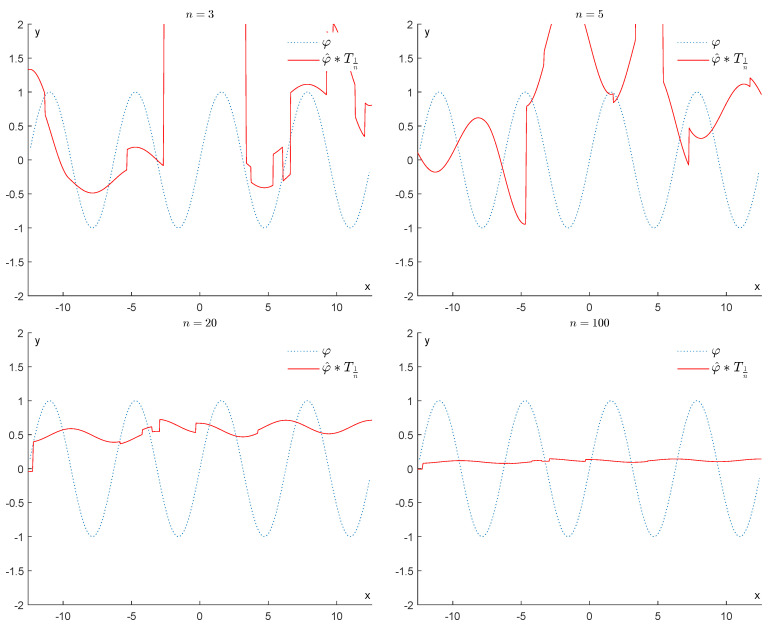
Example 1: Denoising via convolution with $T_{h}$ ($h=\frac{1}{n}$).

If we apply a convolution with $T_{\frac{1}{n}}$, for 3≤n≤100, we obtain the results displayed in Figure 7, showing that all information represented by the function φ is progressively destroyed.

**Figure 8:**
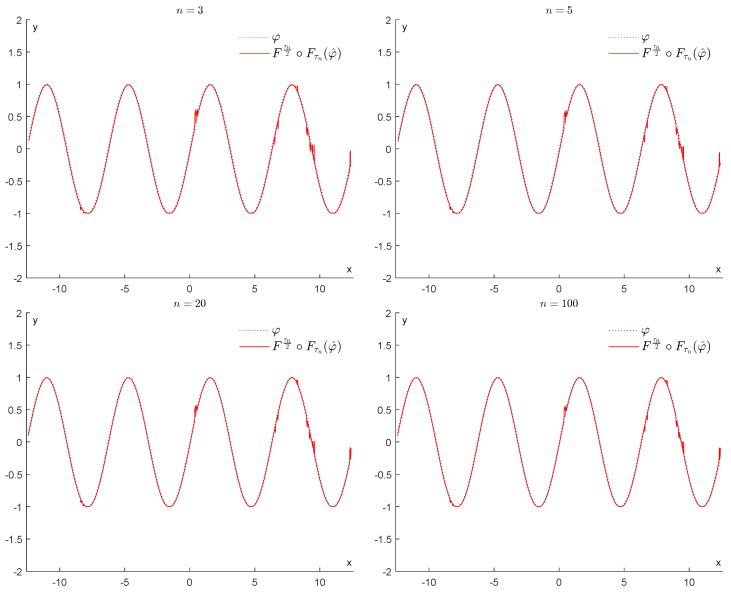
Example 1: Denoising via the proposed GENEO $F^{\frac{\tau_{n}}{2}}\circ F_{\tau_{n}}$.

In contrast, if we apply the operator $F^{\frac{\tau_{n}}{2}}\circ F_{\tau_{n}}\left(\hat{\varphi }\right)$, for n=3,5,20,100, we obtain the results displayed in Figure 8. As we can see, this operator is much more efficient in removing the bumps and restoring the function φ. We recall that as *n* increases, $\tau_{n}$ tends to $2\frac{\sigma }{\beta }$, and our operator becomes more effective at removing the noise.

**Figure 9:**
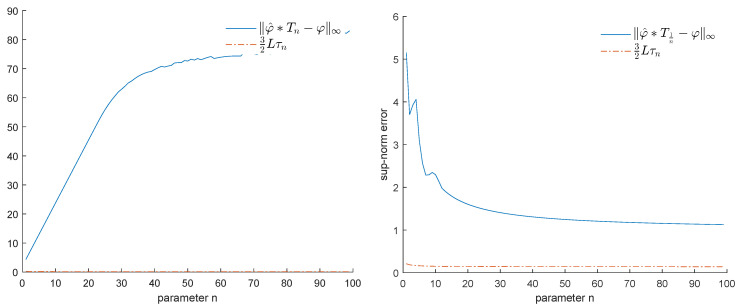
Example 1: Error made by applying a convolution with $T_{n}$ (**left**) or $T_{\frac{1}{n}}$ (**right**).

As a matter of fact, when we apply a convolution with the function $T_{h}$ and check the corresponding errors via the sup-norm, we obtain the results displayed in Figure 9. Since $\lim_{h\to +\infty }\hat{\varphi }*T_{h}=\hat{\varphi }$ and $\lim_{h\to +\infty }\hat{\varphi }*T_{\frac{1}{h}}=0$, we obtain that the errors tend to $∥\hat{\varphi }{-\varphi ∥}_{\infty }=\max_{i=1,\ldots ,N}\left|a_{i}\right|=\bar{\alpha }$ and ${∥\varphi ∥}_{\infty }$, respectively.

**Figure 10:**
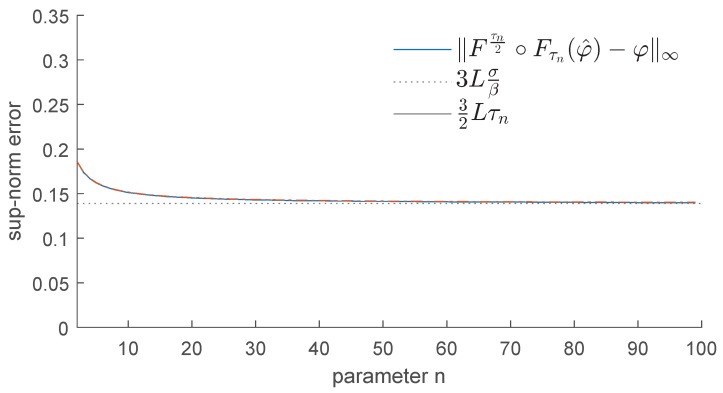
Example 1: Error made by using the GENEO $F^{\frac{\tau_{n}}{2}}\circ F_{\tau_{n}}$.

**Figure 11:**
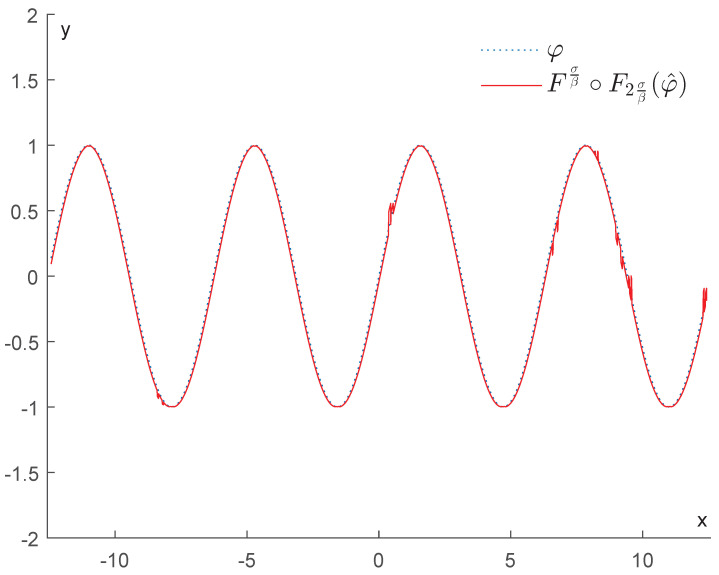
Example 1: Denoising via $F^{\frac{\sigma }{\beta }}\circ F_{2\frac{\sigma }{\beta }}$.

In contrast, if we apply the operator $F^{\frac{\tau_{n}}{2}}\circ F_{\tau_{n}}$, we obtain the results displayed in Figure 10. This shows that the upper bound for the error stated in Corollary 1 is quite tight. As we can expect, the best denoising is achieved when we replace $\tau_{n}$ with $2\frac{\sigma }{\beta }$ (see Figure 11).

**Figure 12:**
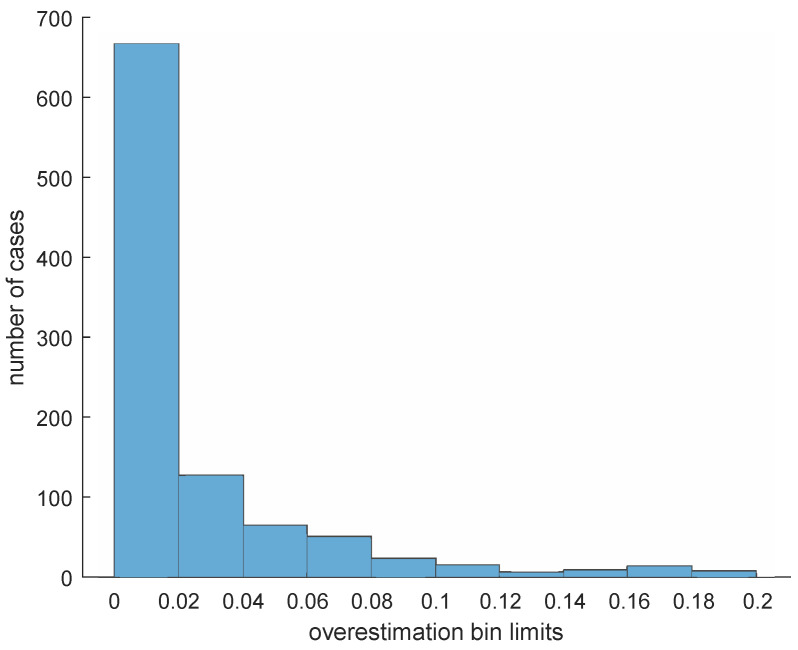
Example 1: Histogram counting the number of cases in each of ten bins concerning the overestimation value $3L\frac{\sigma }{\beta }-{∥F^{\frac{\sigma }{\beta }}\circ F_{2\frac{\sigma }{\beta }}\left(\hat{\varphi }\right)-\varphi ∥}_{\infty }$ obtained in our simulations. Most of the cases belong to the first bin, specifically 665 cases, containing the functions φ for which $0\leq 3L\frac{\sigma }{\beta }-{∥F^{\frac{\sigma }{\beta }}\circ F_{2\frac{\sigma }{\beta }}\left(\hat{\varphi }\right)-\varphi ∥}_{\infty }\leq 0.02$.

Finally, we executed 1000 simulations. In each simulation, we added random impulsive noise to the function φ to produce a function $\hat{\varphi }$. We then applied $F^{\frac{\sigma }{\beta }}\circ F_{2\frac{\sigma }{\beta }}$ to $\hat{\varphi }$ to see how close the upper bound in Corollary 1 is to the actual error $∥F^{\frac{\sigma }{\beta }}\circ F_{2\frac{\sigma }{\beta }}\left(\hat{\varphi }\right){-\varphi ∥}_{\infty }$. The same parameters as in the beginning of this example were used. As we can see in Figure 12, the overestimation committed by our upper bound is often quite close to zero relative to the Lipschitz constant *L* of the function φ.

This suggests that this upper bound is quite accurate.

#### 6.1.2. Second Example

**Figure 13:**
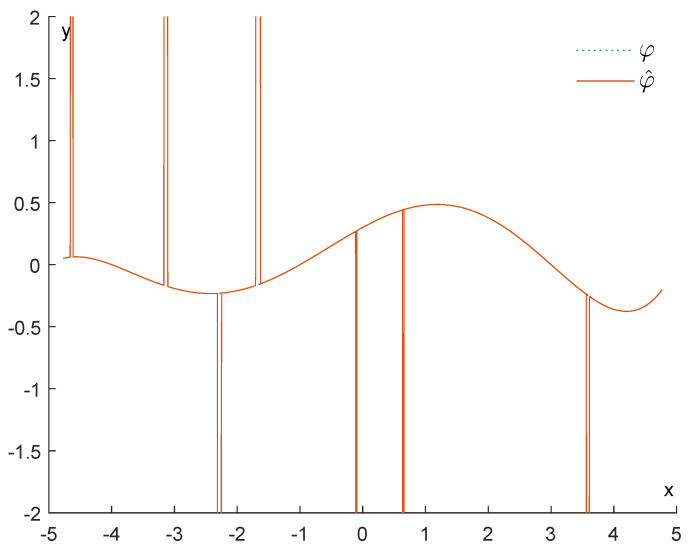
Example 2: Comparison between φ and $\hat{\varphi }$.

Let us consider the function

$$\varphi \left(x\right):=\left\{\begin{array}{cc} \frac{1}{1000}\left(x-5\right)\left(x-3\right)\left(x+1\right)\left(x+4\right)\left(x+5\right) & if-5\leq x\leq 5\\ 0 & otherwise \end{array}\right.$$

for x∈R. The coefficient $\frac{1}{1000}$ was chosen so that the Lipschitz constant *L* would be comparable to the one in the previous example. In this example, $L=\frac{27}{25}$. Figure 13 illustrates how the function $\hat{\varphi }$ looks compared to φ.

**Figure 14:**
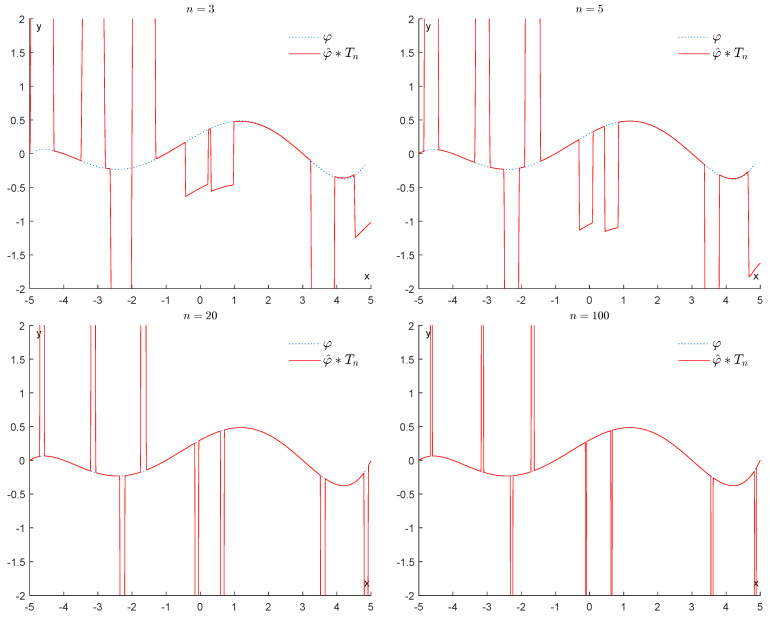
Example 2: Denoising via convolution with $T_{h}$ (h=n).

We will start by considering how well the convolution $\hat{\varphi }*T_{n}$ can approximate the original function φ, when *n* goes from 3 to 100. From Figure 14, it is immediately apparent that the max-norm distance between $\hat{\varphi }*T_{n}$ and φ remains quite large.

**Figure 15:**
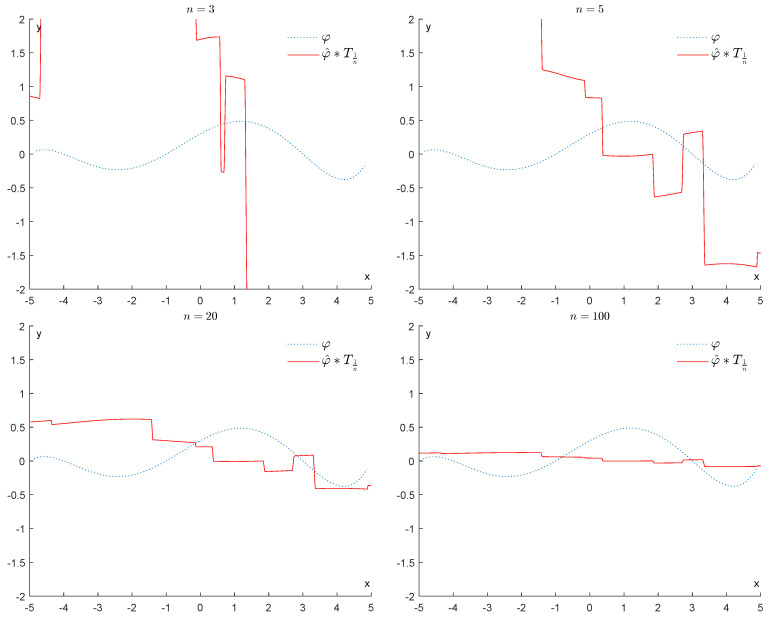
Example 2: Denoising via convolution with $T_{h}$ ($h=\frac{1}{n}$).

If we apply a convolution with $T_{\frac{1}{n}}$, for 3≤n≤100, we obtain the results displayed in Figure 15, showing that all information represented by the function φ is progressively destroyed.

**Figure 16:**
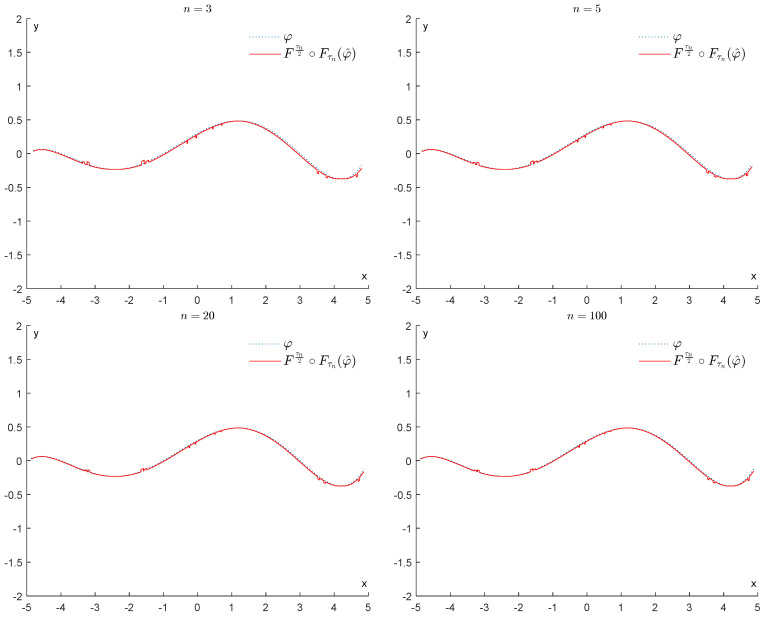
Example 2: Denoising via the proposed GENEO $F^{\frac{\tau_{n}}{2}}\circ F_{\tau_{n}}$.

In contrast, if we apply the operator $F^{\frac{\tau_{n}}{2}}\circ F_{\tau_{n}}\left(\hat{\varphi }\right)$, for n=3,5,20,100, we obtain the results displayed in Figure 16. As we can see, this operator is much more efficient in removing the bumps and restoring the function φ.

**Figure 17:**
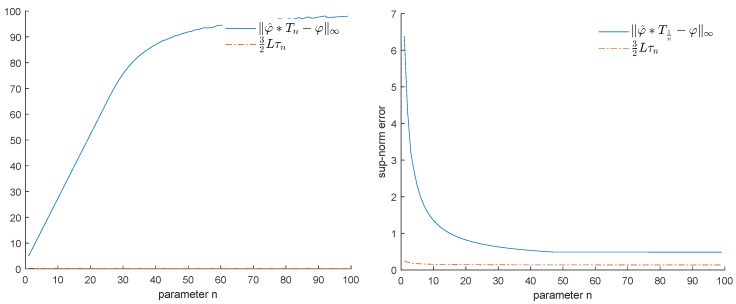
Example 2: Error made by applying a convolution with $T_{n}$ (**left**) or $T_{\frac{1}{n}}$ (**right**).

When we apply a convolution with the function $T_{h}$ and check the corresponding errors via the sup-norm, we obtain the results displayed in Figure 17. As we have already seen, since $\lim_{h\to +\infty }\hat{\varphi }*T_{h}=\hat{\varphi }$ and $\lim_{h\to +\infty }\hat{\varphi }*T_{\frac{1}{h}}=0$, we obtain that the errors tend to $∥\hat{\varphi }{-\varphi ∥}_{\infty }=\max_{i=1,\ldots ,k}\left|a_{i}\right|=\bar{\alpha }$ and ${∥\varphi ∥}_{\infty }$, respectively.

**Figure 18:**
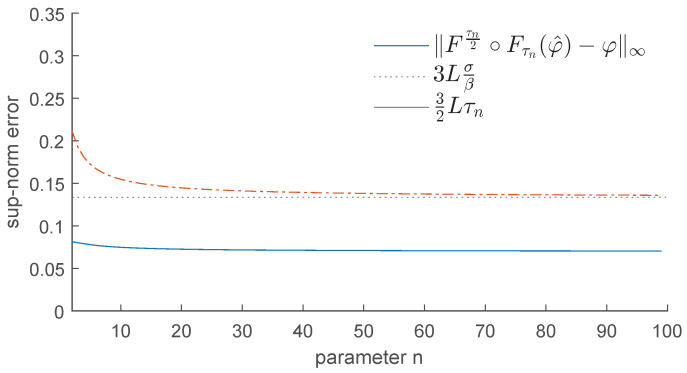
Example 2: Error made by using the GENEO $F^{\frac{\tau_{n}}{2}}\circ F_{\tau_{n}}$.

**Figure 19:**
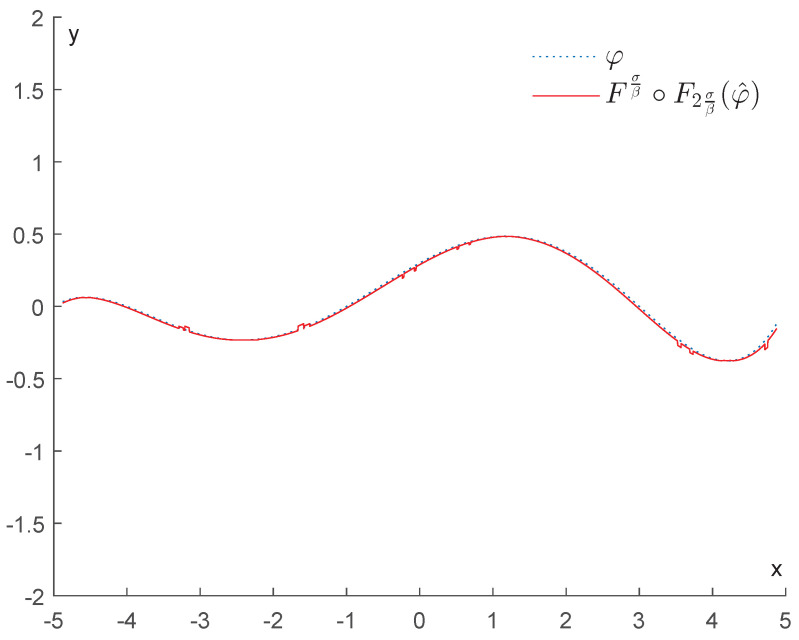
Example 2: Denoising via $F^{\frac{\sigma }{\beta }}\circ F_{2\frac{\sigma }{\beta }}$.

In contrast, if we apply the operator $F^{\frac{\tau_{n}}{2}}\circ F_{\tau_{n}}$, we obtain the results displayed in Figure 18. This shows that the upper bound for the error stated in Corollary 1 is quite tight. As we can expect, the best denoising is achieved when we replace $\tau_{n}$ with $2\frac{\sigma }{\beta }$ (see Figure 19).

**Figure 20:**
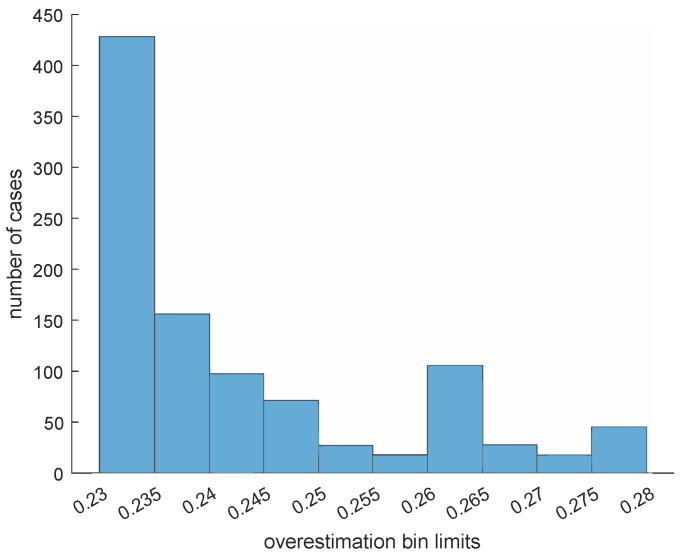
Example 2: Histogram counting the number of cases in each of ten bins concerning the overestimation value $3L\frac{\sigma }{\beta }-{∥F^{\frac{\sigma }{\beta }}\circ F_{2\frac{\sigma }{\beta }}\left(\hat{\varphi }\right)-\varphi ∥}_{\infty }$ obtained in our simulations: 431 cases belong to the first bin and 154 belong to the second one; hence, most of the cases belong to the first two bins containing the functions φ for which $0\leq 3L\frac{\sigma }{\beta }-{∥F^{\frac{\sigma }{\beta }}\circ F_{2\frac{\sigma }{\beta }}\left(\hat{\varphi }\right)-\varphi ∥}_{\infty }\leq 0.24$.

Finally, we again executed 1000 simulations. We used the same methodology as in the previous case, but this time we considered the polynomial presented at the beginning of this example. As we can see in Figure 20, the overestimation committed by our upper bound is often quite close to zero relative to the Lipschitz constant *L* of the polynomial φ.

### 6.2. Experiments

In order to check how good the upper bound stated in Theorem 3 is, we have made the following experiment. In the first step, we fixed ℓ=20 and σ=1.1, and assumed the following parameters to be given: L>0, N∈N, α>0, β>0, k∈N, $a_{1},\ldots ,a_{k}\in ]0,\alpha [$, $b_{1},\ldots ,b_{k}>\beta$, $c_{1},\ldots ,c_{k}\in ]0,\ell [$.

Firstly, we used the parameters L>0, N∈N to generate a random *L*-Lipschitz function φ in the following way. We randomly chose and sorted in ascending order *N* points $x_{1},\ldots ,x_{N}$ in the open interval ]0,ℓ[, with uniform distribution. Hence, we obtained the following decomposition: $]0,\ell \left[=\right]0,x_{1}]\cup ]x_{1},x_{2}]\cup \ldots \cup ]x_{N},\ell [$. We defined our Lipschitz function φ to be 0 outside ]0,ℓ[. After setting $\left(x_{0},y_{0}\right)=\left(0,0\right)$ and $\left(x_{N+1},y_{N+1}\right)=\left(\ell ,0\right)$ for i∈{1,…,N+1}, the value $y_{i}$ of the function at $x_{i}$ was randomly chosen, with uniform distribution in an interval that allows for an *L*-Lipschitz extension to [0,ℓ] of the function, i.e.,

$$\left[\max\left\{y_{i-1}-L\left(x_{i}-x_{i-1}\right),-L\left(\ell -x_{i}\right)\right\},\min\left\{y_{i-1}+L\left(x_{i}-x_{i-1}\right),L\left(\ell -x_{i}\right)\right\}\right].$$

Finally, the graph of the Lipschitz function φ on [0,ℓ] was obtained by connecting each point $\left(x_{i-1},y_{i-1}\right)$ to $\left(x_{i},y_{i}\right)$ with a segment for i∈{1,…,N+1}. We observe that φ constructed this way is an *L*-Lipschitz function.

Secondly, we used the parameters α>0, β>0, k∈N, $a_{1},\ldots ,a_{k}\in ]0,\alpha [$, $b_{1},\ldots ,b_{k}>\beta$, $c_{1},\ldots ,c_{k}\in ]0,\ell [$ to generate a noise function as follows. We considered the mother function ψ defined by setting $\psi \left(x\right):=e^{1-\frac{1}{1-x^{2}}}$ for $x\in ]-1,1[$ and ψ(x):=0 for $x\notin ]-1,1[$. For each *L*-Lipschitz function φ produced in the previously described way, we considered the function $\hat{\varphi }=\varphi +\sum_{i=1}^{k}a_{i}\psi \left(b_{i}\left(x-c_{i}\right)\right)$.

**Figure 21:**
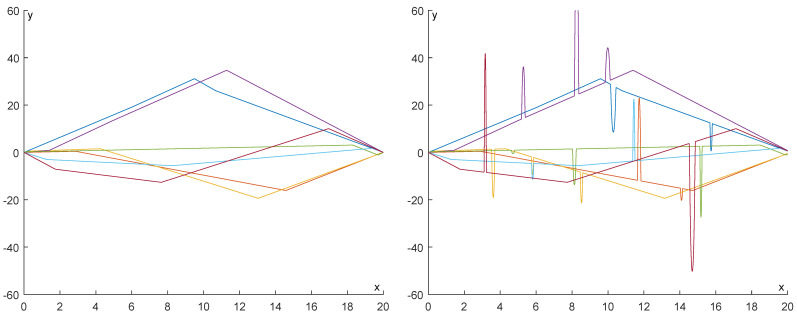
Five examples of the functions we produced for N=3. In each figure, the functions are displayed without noise (**left**) and with added noise (**right**).

**Figure 22:**
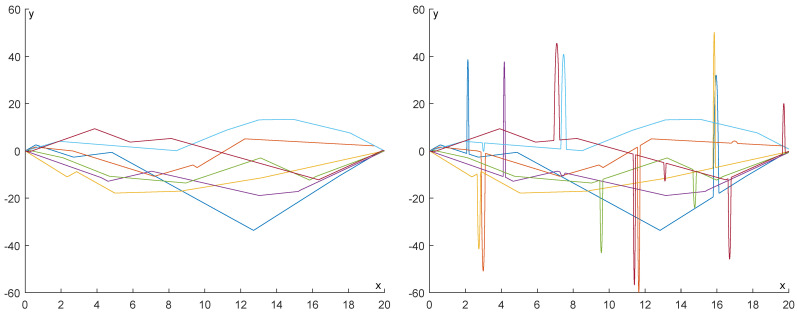
Five examples of the functions we produced for N=7. In each figure, the functions are displayed without noise (**left**) and with added noise (**right**).

In Figure 21 and Figure 22, some examples of the functions we have produced are displayed for N=3 and N=7, respectively. In each figure, the functions are displayed without noise (left) and with added noise (right).

In the second step, we fixed ℓ=20 and σ=1.1 again, and considered a probabilistic model where α, β, *L* are given and the values *N*, *k*, $a_{i}$, $b_{i}$, $c_{i}$ are random variables. In this setting, we compared the noise $∥\hat{\varphi }{-\varphi ∥}_{\infty }$ with the probabilistic upper bound stated in Theorem 3 and the value ${∥F^{\frac{\sigma }{\beta }}\circ F_{2\frac{\sigma }{\beta }}\left(\hat{\varphi }\right)-\varphi ∥}_{\infty }$, which represents the reduced noise that we can obtain by applying our method. In order to average our results, for each triplet (α,β,L) with α∈{50,55,60,…,100}, β∈{3,4,5,…,13}, and L∈{1,2,…,10}, we randomly generated 100 examples of an *L*-Lipschitz function φ and its noisy version $\hat{\varphi }$ by randomly choosing the parameters *N*, *k*, $a_{i}$, $b_{i}$, $c_{i}$ according to the following distributions:

- $N∼{\mathrm{Unif}}_{\left\{1,\ldots ,10\right\}}$
- $k∼{\mathrm{Unif}}_{\left\{1,\ldots ,10\right\}}$
- $a_{i}∼{\mathrm{Unif}}_{]0,\alpha [}$ for i=1,…,k
- $b_{i}∼{\mathrm{Unif}}_{]\beta ,20[}$ for i=1,…,k
- $c_{i}∼{\mathrm{Unif}}_{]3\frac{\sigma }{\beta },\ell -3\frac{\sigma }{\beta }[}$ for i=1,…,k.

**Figure 23:**
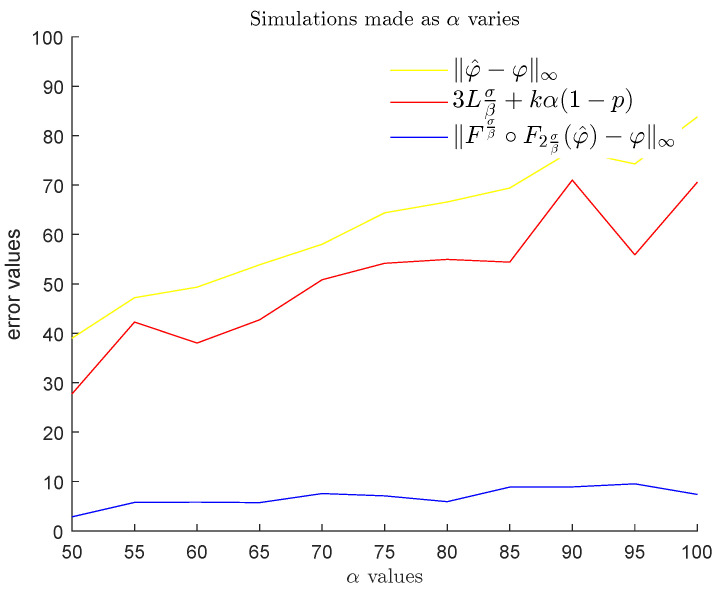
Plots of the means of the averaged values of $∥\hat{\varphi }{-\varphi ∥}_{\infty }$ (yellow), the means of $3L\frac{\sigma }{\beta }+k\alpha \left(1-{\left(1-8\frac{\left(k-1\right)}{\ell }\frac{\sigma }{\beta }\right)}^{k}\right)$ (brown), and the means of the averaged values of ${∥F^{\frac{\sigma }{\beta }}\circ F_{2\frac{\sigma }{\beta }}\left(\hat{\varphi }\right)-\varphi ∥}_{\infty }$ (blue) for β∈{3,4,5,…,13} and L∈{1,2,…,10}, when α varies in the set {50,55,60,…,100}.

**Figure 24:**
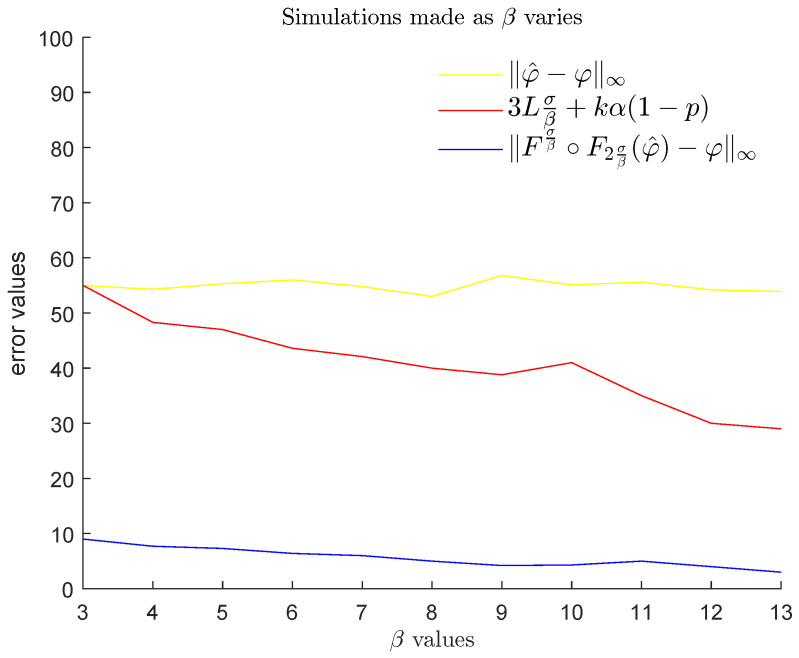
Plots of the means of the averaged values of $∥\hat{\varphi }{-\varphi ∥}_{\infty }$ (yellow), the means of $3L\frac{\sigma }{\beta }+k\alpha \left(1-{\left(1-8\frac{\left(k-1\right)}{\ell }\frac{\sigma }{\beta }\right)}^{k}\right)$ (brown), and the means of the averaged values of ${∥F^{\frac{\sigma }{\beta }}\circ F_{2\frac{\sigma }{\beta }}\left(\hat{\varphi }\right)-\varphi ∥}_{\infty }$ (blue) for α∈{50,55,60,…,100} and L∈{1,2,…,10}, when β varies in the set {3,4,5,…,13}.

**Figure 25:**
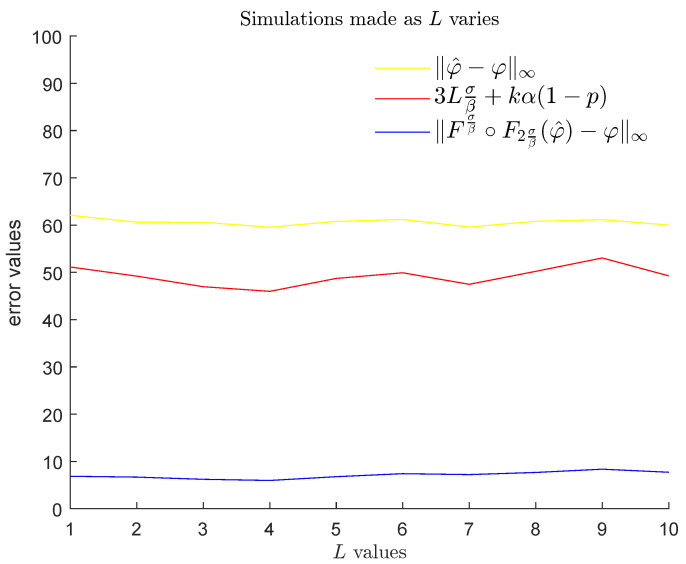
Plots of the means of the averaged values of $∥\hat{\varphi }{-\varphi ∥}_{\infty }$ (yellow), the means of $3L\frac{\sigma }{\beta }+k\alpha \left(1-{\left(1-8\frac{\left(k-1\right)}{\ell }\frac{\sigma }{\beta }\right)}^{k}\right)$ (brown), and the means of the averaged values of ${∥F^{\frac{\sigma }{\beta }}\circ F_{2\frac{\sigma }{\beta }}\left(\hat{\varphi }\right)-\varphi ∥}_{\infty }$ (blue) for α∈{50,55,60,…,100} and β∈{3,4,5,…,13}, when *L* varies in the set {1,2,…,10}.

Then, for the chosen values of each one of the three variables α,β,L, we computed the mean, with respect to the other two variables, of the average of the noise $∥\hat{\varphi }{-\varphi ∥}_{\infty }$ and the average of the reduced noise ${∥F^{\frac{\sigma }{\beta }}\circ F_{2\frac{\sigma }{\beta }}\left(\hat{\varphi }\right)-\varphi ∥}_{\infty }$ obtained by our method, both evaluated for $\left(\varphi ,\hat{\varphi }\right)$ varying in the set of cardinality 100 that we produced. We also computed the mean of the probabilistic upper bound stated in Theorem 3 with respect to the same two variables. The results are displayed in Figure 23, Figure 24 and Figure 25. We remind the reader that α and β, respectively, express the maximum height and the thinness of the noise bumps, while *L* is a Lipschitz constant for each function φ that we are interested in. These results illustrate the effectiveness of using GENEOs in the reduction of impulsive noise.

## 7. Conclusions

In our paper, we have proved a stability property for persistence diagrams of functions from R to R in the presence of impulsive noise. This property shows that TDA can also be useful when noise drastically changes the topology of the sublevel sets of the filtering functions we are considering, and highlights some new potential interactions between TDA and the theory of GENEOs. The experimental section shows that our approach is indeed able to remove impulsive noise in most cases. It would be interesting to investigate the possibility of extending our method to real-valued functions defined on *n*-dimensional domains and allowing the function ψ to be a random variable, by selecting suitable GENEOs. We observe that the operators $F^{\varepsilon }$ and $F_{\varepsilon }$ introduced in Definition 3 can be easily adapted to the case of functions from $R^{n}$ to R, by setting $F^{\varepsilon }\left(\varphi \right)\left(x\right)=\max\{\varphi \left(x+v\right)|v\in R^{n},∥v∥=\varepsilon \}$ and $F_{\varepsilon }\left(\varphi \right)\left(x\right)=\min\{\varphi \left(x+v\right)|v\in R^{n},∥v∥=\varepsilon \}$ for all $x\in R^{n}$. Applying a suitable operator $F^{\delta }\circ F_{\varepsilon }$ to real-valued filtering functions defined on $R^{n}$ should allow us to control the expected value of the perturbation of persistence diagrams caused by uniformly distributed impulsive noise, even for n>1 and in degrees greater than 0. Our long-term goal is to realize Machine Learning methods in which optimal operators for impulse noise removal can be learned and selected in the convex hull of a given finite set of GENEOs, using a theorem which guarantees that the convex combination of GENEOs is still a GENEO (cf. [11]). We plan to devote our research to this topic in the future.

## Data Availability

Data available on request from the authors.

## References

[1] Edelsbrunner H., Morozov D. (2013). Persistent homology: Theory and practice. European Congress of Mathematics.

[2] Biasotti S., Floriani L.D., Falcidieno B., Frosini P., Giorgi D., Landi C., Papaleo L., Spagnuolo M. (2008). Describing shapes by geometrical-topological properties of real functions. ACM Comput. Surv..

[3] Carlsson G. (2009). Topology and data. Bull. Amer. Math. Soc..

[4] Edelsbrunner H., Harer J. (2008). Persistent homology—A survey. Surveys on Discrete and Computational Geometry.

[5] Cohen-Steiner D., Edelsbrunner H., Harer J. (2007). Stability of persistence diagrams. Discrete Comput. Geom..

[6] Cohen-Steiner D., Edelsbrunner H., Harer J., Mileyko Y. (2010). Lipschitz functions have Lp-stable persistence. Found. Comput. Math..

[7] Fasy B.T., Lecci F., Rinaldo A., Wasserman L., Balakrishnan S., Singh A. (2014). Confidence sets for persistence diagrams. Ann. Stat..

[8] Buchet M., Chazal F., Dey T.K., Fan F., Oudot S.Y., Wang Y., Arge L., Pach J. (2015). Topological analysis of scalar fields with outliers. Proceedings of the 31st International Symposium on Computational Geometry (SoCG 2015).

[9] Adler R.J., Agami S. (2019). Modelling persistence diagrams with planar point processes, and revealing topology with bagplots. J. Appl. Comput. Topol..

[10] Vishwanath S., Fukumizu K., Kuriki S., Sriperumbudur B.K., Larochelle H., Ranzato M., Hadsell R., Balcan M.F., Lin H. (2020). Robust persistence diagrams using reproducing kernels. Advances in Neural Information Processing Systems.

[11] Bergomi M.G., Frosini P., Giorgi D., Quercioli N. (2019). Towards a topological–geometrical theory of group equivariant non-expansive operators for data analysis and machine learning. Nat. Mach. Intell..

[12] Conti F., Frosini P., Quercioli N. (2022). On the construction of Group Equivariant Non-Expansive Operators via permutants and symmetric functions. Front. Artif. Intell..

[13] Bocchi G., Botteghi S., Brasini M., Frosini P., Quercioli N. (2023). On the finite representation of linear group equivariant operators via permutant measures. Ann. Math. Artif. Intell..

[14] Frosini P., Jabłoński G. (2016). Combining persistent homology and invariance groups for shape comparison. Discrete Comput. Geom..

[15] Cerri A., Fabio B.D., Ferri M., Frosini P., Landi C. (2013). Betti numbers in multidimensional persistent homology are stable functions. Math. Methods Appl. Sci..

[16] Cerri A., Ethier M., Frosini P. (2019). On the geometrical properties of the coherent matching distance in 2D persistent homology. J. Appl. Comput. Topol..

[17] Micheletti A. (2023). A new paradigm for artificial intelligence based on group equivariant non-expansive operators. Eur. Math. Soc. Mag..

[18] Vaseghi S.V. (2008). Impulsive Noise: Modelling, Detection and Removal.

[19] Earnest M. Average Minimum Distance between *n* Points Generate i.i.d. with Uniform Dist. Mathematics Stack Exchange. https://math.stackexchange.com/q/2001026.

